# *Seco*-tremulane Sesquiterpenoids from the Cultures of the Medicinal Fungus *Irpex lacteus* HFG1102

**DOI:** 10.1007/s13659-018-0157-y

**Published:** 2018-03-19

**Authors:** He-Ping Chen, Zhen-Zhu Zhao, Zheng-Hui Li, Tao Feng, Ji-Kai Liu

**Affiliations:** School of Pharmaceutical Sciences, South-Central University for Nationalities, Wuhan, 430074 People’s Republic of China

**Keywords:** *Irpex lacteus*, Meruliaceae, 5,6-*seco*-tremulane, Sesquiterpenoid

## Abstract

**Abstract:**

Six previously undescribed 5,6-*seco*-tremulane analogues, together with two known ones, were isolated from the culture broth of the medicinal fungus *Irpex lacteus* HFG1102. The structures of the new compounds were elucidated via extensive spectroscopic methods, including NMR and HRMS spectroscopic analyses.

**Graphical Abstract:**

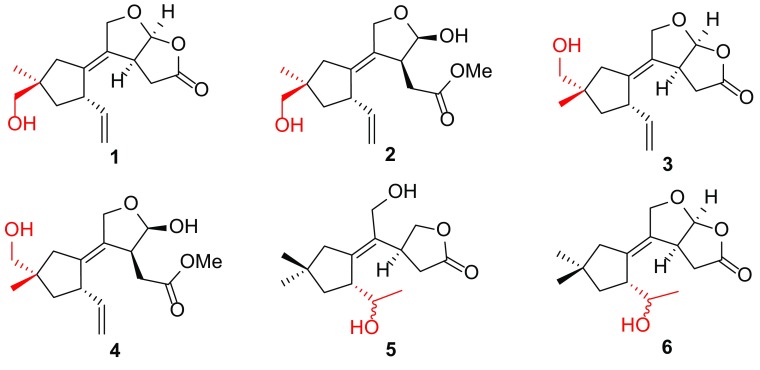

**Electronic supplementary material:**

The online version of this article (10.1007/s13659-018-0157-y) contains supplementary material, which is available to authorized users.

## Introduction

Higher fungi are valuable and irreplaceable resources of natural product-derived lead compounds for drug research and development [1]. The fungus *Irpex lacteus* has been used as a medicinal fungus for decades in China for the treatment of inflammation, bacterial/fungal infection, and urinary retention [2]. The polysaccharide products of the submerged culture of *I. lacteus* have been developed and approved by SFDA as a clinically used drug for curing chronic glomerulonephritis [2]. However, although this fungus has been widely used as an industrial source to obtain polysaccharide products, its secondary metabolites remained unknown.

**Fig. 1:**
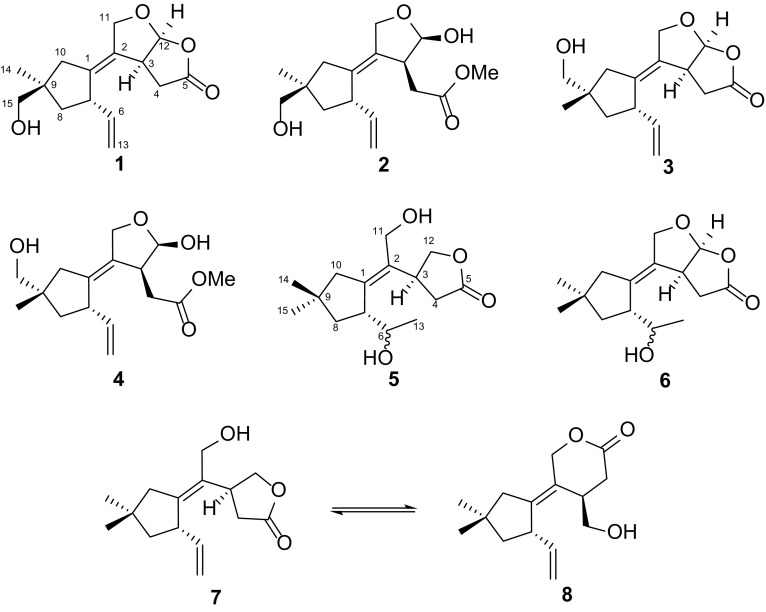
Structures of compounds **1**–**8**

The 5,6-*seco*-tremulane skeleton, which was regarded as the resultant of Baeyer–Villiger oxidation of tremulane, was first and mainly reported from the basidiomycete *Conocybe siliginea* [3, 4]. To the best of our knowledge, the distribution of this type of sesquiterpenes only reported from three species, the basidiomycete *Flavodon flavus* BCC 17421 [5], and the endophytic fungi *Colletotrichum capsici* [6] and *Ceriporia lacerate* [7]. Notably, this type of sesquiterpenes always presented in inseparable mixtures. This study, as part of our ongoing search for promising lead compounds from higher fungi, concentrated on the secondary metabolites of the culture broth of the medicinal fungus *I. lacteus*, led to the isolation of six undescribed 5,6-*seco*-tremulane congeners (**1**–**6**) and two known tautomeric isomers (**7**, **8**) (Fig. 1). Herein, we report the isolation, structure elucidation of the new compounds.

## Results and Discussion

Compounds **1** and **2** were obtained as an inseparable colourless mixture with the ratio of approximately 1:0.9 deduced from the intensity of ^1^H NMR resonance. The ^1^H and ^13^C NMR spectra of the mixture displayed paired signals, indicating the similar structures of **1** and **2**. Nevertheless, comprehensive analysis of the 1D and 2D NMR spectra expedited the structural elucidation of **1** and **2** unambiguously based on the relatively less overlapped signals. Elucidating of the HSQC, HMBC, and ^1^H–^1^H COSY spectra facilitated the assignments of two class of signals corresponding to **1** and **2**.

**Fig. 2:**
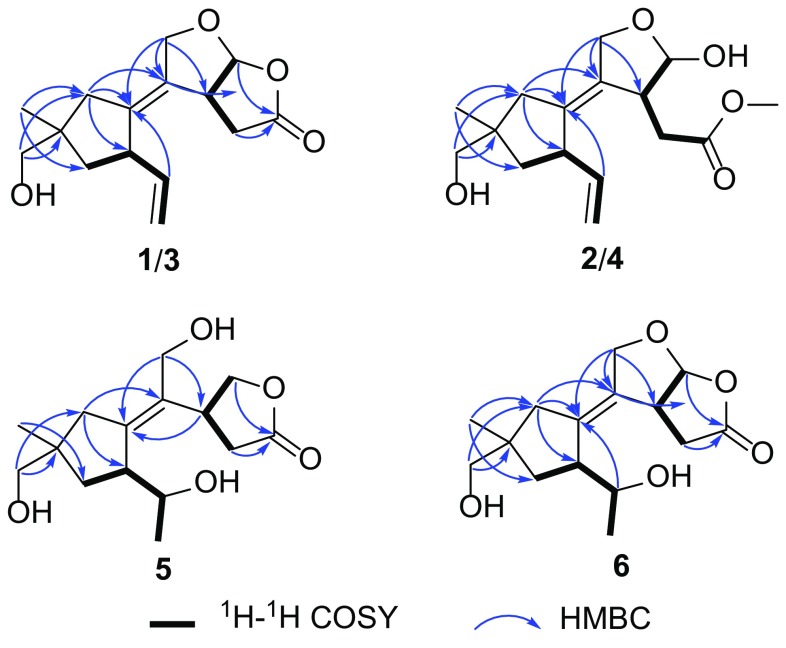
Key ^1^H–^1^H COSY and HMBC correlations of compounds **1**–**6**

The signals assigned for compound **1** consisted of a methyl singlet, six methylenes, four methines (including a ketal carbon), and four quaternary carbons (two olefinic, one carbonyl) (Tables 1, 2). The ^1^H–^1^H COSY of H-13/H-6/H-7/H-8, and H-4/H-3/H-12 enabled the connection of C-13–C-6–C-7–C-8 and C-12–C-3–C-4, respectively. The HMBC correlations from H_3_-14 (*δ*_H_ 1.09) and H-15 (*δ*_H_ 3.22, 3.19) to C-9 (*δ*_C_ 44.6), C-10 (*δ*_C_ 42.7), and C-8 (*δ*_C_ 44.4), along with from H-10 (*δ*_H_ 2.31, 2.00) to C-1 (*δ*_C_ 139.4), C-2 (*δ*_C_ 133.2), and C-7 (*δ*_C_ 49.3) enabled the completion of a five-membered ring constructed by C-1–C-7–C-8–C-9–C-10 and an exocyclic double bond located at C-1 (Part A) (Fig. 2). Moreover, the HMBC correlations from oxygen-bearing methylene H-11 (*δ*_H_ 4.49, 4.39) to C-1, C-2, C-3 (*δ*_C_ 42.4), and C-12 (*δ*_C_ 110.6), and from H-12 (*δ*_H_ 6.09) and H-4 (*δ*_H_ 2.92, 2.49) to the carbonyl C-5 (*δ*_C_ 178.3) led to construction of tetrahydrofuro[2,3-*b*]furan-2(3*H*)-one moiety which connected with part A through the double bond C-1–C-2 (Fig. 2). The above evidences led to the establishment of the planar structure of **1**, which also in accordance with the molecular formula deduced from HRESIMS result which gave a sodium adduct ion peak at *m/z* 287.1258 [M+Na]^+^ (calcd for C_15_H_20_O_4_Na, 287.1254).

**Table 1:** ^1^H NMR spectroscopic data of compounds **1**–**6** (*δ* in ppm)

No.	**1** ^a^	**2** ^a^	**3** ^a^	**4** ^a^	**5** ^a^	**6** ^b^
3	3.70, m	3.03, dd (11.1, 2.5)	3.74, m	3.07, dd (11.4, 2.8)	3.87, m	3.78, m
4	2.92, dd (19.0, 11.0) 2.49, dd (19.0, 4.1)	2.56, dd (16.5, 2.5) 2.25, dd (16.5, 11.1)	2.94, dd (19.2, 11.0) 2.49, dd (19.2, 4.0)	2.56, dd (16.6, 2.8) 2.26, dd (16.6, 11.4)	2.63, dd (17.6, 9.4) 2.57, dd (17.6, 9.3)	2.90, dd (17.8, 9.6) 2.65, dd (17.8, 2.0)
6	5.78, ddd (17.0, 9.7, 7.2)	5.75, ddd (17.8, 9.5, 9.5)	5.73, ddd (17.0, 9.7, 7.2)	5.74, dd (17.8, 9.5, 9.5)	3.70, qd (6.0, 6.0)	4.21, m
7	3.35, m	3.35, m	3.43, m	3.44, m	2.95, m	2.82, m
8	1.97, overlapped 1.31, dd (13.2, 8.5)	1.97, overlapped 1.34, dd (13.4, 8.5)	1.67, dd (12.7, 8.2) 1.55, dd (12.7, 9.3)	1.64, dd (12.4, 8.3) 1.54, dd (12.4, 8.7)	1.61, dd (13.2, 8.9) 1.38, dd (13.2, 6.9)	1.62, dd (12.9, 10.4) 1.58, dd (12.9, 8.3)
10	2.31, br d (16.2) 2.00, br d (16.2)	2.19, br d (16.2) 1.94, br d (16.2)	2.22, d (14.3) 1.99, d (14.3)	2.16, br d (14.9) 1.88, br d (14.9)	2.31, br d (14.5) 2.02, br d (14.5)	2.03, br d (16.8) 1.83, br d (16.8)
11	4.49, d (12.4) 4.39, d (12.4)	4.41, br d (12.1) 4.24, br d (12.1)	4.47, d (11.6) 4.35, d (11.6)	4.40, br d (12.1) 4.24, br d (12.1)	4.22, d (12.1) 4.16, d (12.1)	4.56, br d (12.3) 4.46, br d (12.3)
12	6.09, d (5.6)	5.19, s	6.10, d (5.7)	5.21, s	4.40, dd (8.8, 8.8) 4.28, dd (8.8, 8.8)	6.20, d (5.5)
13	5.09, dd (17.0, 1.3) 5.00, dd (9.7, 1.3)	5.06, br d (17.8) 4.90, overlapped	5.12, dd (17.0, 1.5) 5.01, dd (9.7, 1.5)	5.09, br d (17.7) 4.90, overlapped	1.09, d (6.0)	1.17, d (7.0)
14	1.09, s	1.09, s	3.41, d (12.0) 3.40, d (12.0)	3.40, d (12.0) 3.39, d (12.0)	1.12, s	1.12, s
15	3.22, d (11.0) 3.19, d (11.0)	3.26, d (12.0) 3.24, d (12.0)	0.92, s	0.95, s	0.86, s	0.85, s
16		3.65, s		3.66, s		

^a^Recorded in CD_3_OD, recorded in 600 MHz

^b^Recorded in CDCl_3_, recorded in 800 MHz

**Table 2:** ^13^C NMR spectroscopic data of compounds **1**–**6** (150 MHz, *δ* in ppm)

No.	**1** ^a^	**2** ^a^	**3** ^a^	**4** ^a^	**5** ^a^	**6** ^b^
1	139.4, C	138.0, C	139.1, C	137.6, C	148.7, C	138.1, C
2	133.2, C	133.8, C	133.5, C	134.1, C	130.6, C	130.0, C
3	42.4, CH	46.6, CH	42.4, CH	46.5, CH	39.3, CH	41.5, CH
4	37.2, CH_2_	37.1, CH_2_	37.3, CH_2_	37.2, CH_2_	34.0, CH_2_	34.5, CH_2_
5	178.3, C	174.3, C	178.3, C	174.3, C	180.5, C	174.3, C
6	144.2, CH	144.5, CH	144.0, CH	144.3, CH	71.4, CH	66.3, CH
7	49.3, CH	48.7, CH	48.3, CH	48.8, CH	49.2, CH	47.9, CH
8	44.4, CH_2_	44.3, CH_2_	44.1, CH_2_	44.2, CH_2_	42.5, CH_2_	39.9, CH_2_
9	44.6, C	44.7, C	44.8, C	44.8, C	38.4, C	37.4, C
10	42.7, CH_2_	42.6, CH_2_	42.9, CH_2_	42.6, CH_2_	46.5, CH_2_	48.5, CH_2_
11	70.2, CH_2_	69.4, CH_2_	70.2, CH_2_	69.5, CH_2_	60.1, CH_2_	71.5, CH_2_
12	110.6, CH	102.7, CH	110.5, CH	102.7, CH	73.9, CH_2_	109.8, CH
13	115.0, CH_2_	114.5, CH_2_	115.1, CH_2_	114.5, CH_2_	19.4, CH_3_	20.7, CH_3_
14	69.2, CH_2_	69.3, CH_2_	23.1, CH_3_	23.2, CH_3_	29.6, CH_3_	27.4, CH_3_
15	24.5, CH_3_	24.5, CH_3_	70.9, CH_2_	71.1, CH_2_	28.6, CH_3_	28.6, CH_3_
16		52.3, CH_3_		52.3, CH_3_		

^a^Recorded in CD_3_OD, recorded in 150 MHz

^b^Recorded in CDCl_3_, recorded in 200 MHz

A ROESY experiment determined the relative configuration of compound **1**. The ROESY cross peaks of H-7/H-8*β*/H-15 led to the assignment of H-7 and 15-CH_2_OH as *β* orientation. The significant ROESY correlation between H-3 and H-12, H-10 and H-11 suggested that H-3 and H-12 were at the same side, and the C-1–C-2 double bond were *Z* configuration. Therefore, the structure of compound **1** was established as 11,12-epoxy-15-hydroxy-5,6-*seco*-tremula-1,6(13)-dien-5,12-olide (Fig. 1).

The other set of signals for compound **2** showed resemblance with those of compound **1**, but with the presence of an additional methoxy group (*δ*_H_ 3.65, *δ*_C_ 52.3). Careful analysis of the 2D NMR led to the conclusion that the only difference between **1** and **2** was the lactone group in **1** opened in **2** and the carbonyl group was methyl esterified, which was confirmed by the HMBC correlation from the methoxy (*δ*_H_ 3.65) to the carbonyl C-5 (*δ*_C_ 174.3) as well as the absence of HMBC correlation from H-12 to C-5. The relative configuration of **2** was identical with that of **1** by interpretation of the ROESY spectrum. The HRESIMS result of the mixture also gave a molecular formula of C_16_H_24_O_5_ (*m/z* 319.1529 [M+Na]^+^, calcd for C_16_H_24_O_5_Na, 319.1516) which further reinforced the above assignment. Thus, compound **2** was deduced as methyl 12*β*,15-dihydroxy-5,6-*seco*-tremula-1,6(13)-dien-5-oate (Fig. 1).

**Fig. 3:**
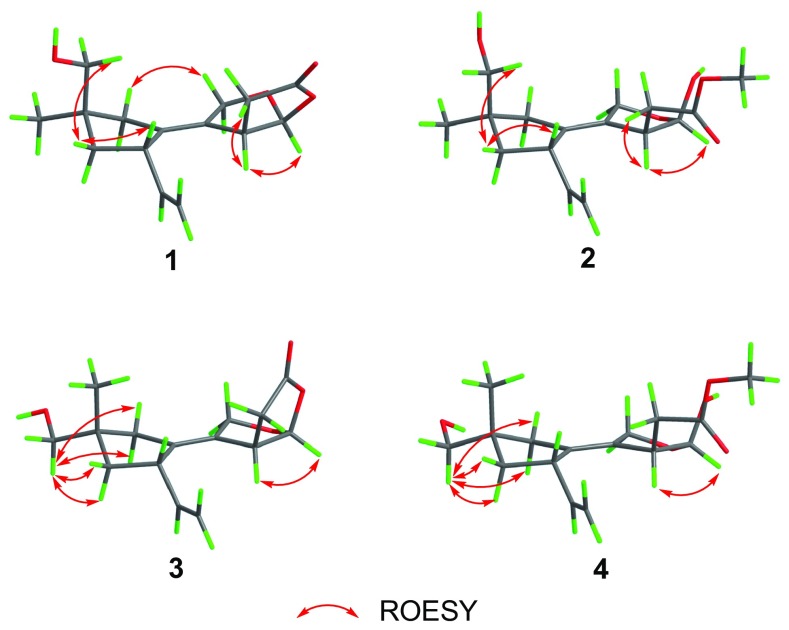
Key ROESY correlations of compounds **1**–**4**

Compounds **3** and **4** were isolated as an inseparable mixture with the ratio of 1:0.6. The 1D NMR data of the mixture showed high similarities with those of the **1**/**2** mixture, implying that compounds **3** and **4** were analogues of **1** and **2**. Elucidating the 2D NMR spectra of the mixture promoted the completion of **3** and **4** which possessing the same planar structures with those of **1** and **2**, respectively. Considering of the above information, a conclusion could be drawn which compounds **3** and **4** differed from **1** and **2** in spatial configuration. Analysis of the ROESY spectrum of **3**/**4**, in combination with ChemBio3D simulations confirmed the speculation. The ROESY spectrum displayed cross peaks of H-14 (*δ*_H_ 3.41, 3.40 for **3**; 3.40, 3.39 for **4**)/H-8 (*δ*_H_ 1.67, 1.55 for **3**; 1.64, 1.54 for **4**), H-14/H-10 (*δ*_H_ 2.22, 1.99 for **3**; 2.16, 1.88 for **4**). As shown in Fig. 3, the minimized energy optimized molecular models of **3** and **4** suggested that only when the 14-CH_2_OH adopted the equatorial direction, the above ROESY correlation could be seen. Therefore, compounds **3** and **4** were C-9 epimers of compounds **1** and **2**, respectively. This conclusion was also evidenced by the large discrimination of ^13^C NMR chemical shifts of C-14 and C-15 between **1** and **3**, **2** and **4**. Thus, compounds **3** and **4** were identified as shown in Fig. 1, and were given the respective names 11,12-epoxy-14-hydroxy-5,6-*seco*-tremula-1,6(13)-dien-5,12-olide, and methyl 12*β*,14-dihydroxy-5,6-*seco*-tremula-1,6(13)-dien-5-oate.

Compound **5** was isolated as colourless oil. The HRESIMS result presented a molecular formula of C_15_H_24_O_4_ as indicated by the sodium adduct ion peak at *m/z* 291.1572 [M+Na]^+^ (calcd for C_15_H_24_O_4_Na, 291.1567). The 1D NMR data of **5** (Tables 1, 2) were classified into three methyls, five methylenes (two oxygenated), three methines (one oxygenated), and four quaternary carbons (two olefinic ones, and a carbonyl). These data closely related to the co-isolate conocenolide A (**7**) [3], suggesting compound **5** was a congener of **7**. The main discrepancy between **5** and **7** was that the absence of the C-6–C-13 double bond while the presence of an oxygen-bearing methine and a methyl doublet in **5** compared to those of **7**, suggested that the C-6 in **7** was oxygenated in **5**, which was verified by the ^1^H–^1^H COSY correlations of H-6/H-7, H-6/H-13 (Fig. 2). Unfortunately, the inadequate sample of **5** (0.8 mg) precluded the determination of the absolute configuration of 6-OH. Therefore, compound **5** was identified as 6,11-dihydroxy-5,6-*seco*-tremul-1-en-5,12-olide (Fig. 1).

Compound **6** was obtained as colourless oil. The ^1^H and ^13^C NMR spectra exhibited signals which ascribable to three methyls, four methylenes (one oxygenated), four methines (two oxygenated), and four quaternary carbons (a tetrasubstituted double bond, a carbonyl). These data were reminiscent of those of compounds **1** and **3** (Tables 1, 2). Compared the NMR data of **6** with those of **1** revealed that the large differences located at C-6, C-13, and C-15. Detailed analysis of the 2D NMR spectra of **6** indicated that the C-6–C-13 double bond was oxygenated, while C-15 remained unoxygenated. This postulation was validated by the ^1^H–^1^H COSY correlations of H-6/H_3_-13, and H-6/H-7, and HMBC correlation from H_3_-14 to H_3_-15 (Fig. 2). Due to scarcity of samples, 6-OH remained unassigned. The above assignments were consistent with the HRESIMS data, which gave a sodium adduct ion peak at *m/z* 289.1416 [M +Na]^+^, suggesting a molecular formula of C_15_H_22_O_4_ (calcd for C_15_H_22_O_4_Na, 289.1410). Thus, the structure of **6** was established as shown in Fig. 1 and was named as 11,12-epoxy-6-hydroxy-5,6-*seco*-tremul-1-en-5,12-olide.

Conocenolides A (**7**) and B (**8**) [3] were two abundant and separable but tautomeric isomers accompanied in this study. Their structures were identified compared with the literature data.

In conclusion, six hitherto unknown 5,6-*seco*-tremulanes were isolated from the culture broth of the medicinal fungus *I. lacteus*. All these 5,6-*seco*-tremulane skeletons were proposed to be biosynthesized via Baeyer–Villiger oxidation, rearrangement, elimination, oxidation, and esterification of the corresponding 5-oxo-tremulane. This study represents the first report of 5,6-*seco*-tremulanes from the fungus *I. lacteus*, and also expands the distribution of 5,6-*seco*-tremulane-type sesquiterpenoids as fungal metabolites.

## Experimental

### General Experimental Procedures

Optical rotations were obtained on a JASCO P-1020 digital polarimeter (Horiba, Kyoto, Japan). UV spectra were recorded on a Shimadzu UV-2401PC UV–Visible recording spectrophotometer (Shimadzu, Kyoto, Japan). A Tenor 27 spectrophotometer (Bruker Optics GmbH, Ettlingen, Germany) was used for scanning IR spectroscopy using KBr pellets. 1D and 2D NMR spectra were obtained on a Bruker Avance III 600 MHz spectrometer (Bruker Corporation, Karlsruhe, Germany). HRESIMS were recorded on an Agilent 6200 Q-TOF MS system (Agilent Technologies, Santa Clara, CA, USA). Sephadex LH-20 (Amersham Biosciences, Uppsala, Sweden) and silica gel (Qingdao Haiyang Chemical Co., Ltd., Qingdao, China) were used for column chromatography (CC). Medium Pressure Liquid Chromatography (MPLC) was performed on a Büchi Sepacore System equipped with pump manager C-615, pump modules C-605 and fraction collector C-660 (Büchi Labortechnik AG, Flawil, Switzerland), and columns packed with Chromatorex C-18 (dimensions 450 mm × i.d. 14 mm, particle size: 40–75 μm, Fuji Silysia Chemical Ltd., Kasugai, Japan). Preparative high performance liquid chromatography (prep-HPLC) were performed on an Agilent 1260 liquid chromatography system equipped with a Zorbax SB-C18 column (particle size 5 μm, dimensions 150 mm × i.d. 9.4 mm, flow rate 7 ml min^−1^) and a DAD detector (Agilent Technologies, Santa Clara, CA, USA).

### Fungal Material

The fungus *Irpex lacteus* was collected from Changbai Mountain Nature Reserve in 2012. The strain of *I. lacteus* in this study was isolated from the fresh fruiting bodies and kept on potato, dextrose, and agar (PDA) culture medium. A voucher specimen (No. CGBWSHFG1102) was deposited in the Herbarium of Kunming Institute of Botany, Chinese Academy of Sciences. The culture medium to ferment this fungus consisted of glucose (5%), peptone from porcine meat (0.15%), yeast powder (0.5%), KH_2_PO_4_ (0.05%) and MgSO_4_ (0.05%). Sixty Erlenmeyer flasks (500 ml) each containing 350 ml of above-mentioned culture medium were inoculated with *I. lacteus* strains, respectively. Fermentation were carried out on rotatory shakers at 25 °C and 150 rpm for 25 days in dark environment.

### Extraction and Isolation

The culture broth (20 l) of *I. lacteus* HFG1102 was filtered and concentrated to 3 l followed by partitioned between EtOAc and water for four times to give an EtOAc layer. Meanwhile, the mycelia were extracted by EtOH (95%) for three times. The EtOAc layer together with the mycelium extract were concentrated under reduced pressure to afford a crude extract (wt 16.0 g). This residue was separated by MPLC (MeOH/H_2_O, 0–100%) to give sixteen main fractions (A–P).

Subfraction I was separated by Sephadex LH-20 CC (MeOH) to afford three subfractions I1–I3. Subfraction I3 was further purified by prep-HPLC (MeCN/H_2_O, 30–50%, 25 min) to give two isolated but interchangeable compounds **7** (*t*_R_ = 16.5 min) and **8** (*t*_R_ = 17.8 min) with a totally yield 22 mg.

Subfraction J was separated by Sephadex LH-20 CC (MeOH) to remove colourants, and further separated by Sephadex LH-20 (acetone) to afford four subfractions J1–J4. Subfraction J2 was purified by prep-HPLC (MeCN/H_2_O, 26–46%, 25 min) to give **1**/**2** (*t*_R_ = 16.5 min) with a yield 2.0 mg. Subfraction J3 was purified by prep-HPLC (MeCN/H_2_O, 26–46%, 25 min) to give **3**/**4** (*t*_R_ = 18.2 min) with a yield 1.8 mg.

Subfraction K was separated by Sephadex LH-20 CC (MeOH) to remove colourants, and further separated by Sephadex LH-20 (acetone) to afford five subfractions K1–K5. Subfraction K1 was purified by prep-HPLC (MeCN/H_2_O, 25–45%, 25 min) to give **5** (*t*_R_ = 17.5 min) with a yield 1.0 mg. Subfraction K3 was purified by prep-HPLC (MeCN/H_2_O, 26–46%, 25 min) to give **6** (*t*_R_ = 18.9 min) with a yield 0.8 mg.

### Spectroscopic Data of Compounds

#### 11,12-Epoxy-15-hydroxy-5,6-*seco*-tremula-1,6(13)-dien-5,12-olide (**1**) and methyl 12*β*,15-dihydroxy-5,6-*seco*-tremula-1,6(13)-dien-5-oate (**2**)

Colourless oil; $[\alpha ]_{{\text{D}}}^{{26}}$ − 56.9 (*c* 0.09, MeOH); UV (*c* 0.0306 mg ml^−1^, MeOH) *λ*_max_ nm (Abs): 205 (0.7693), 232 (0.3060); IR (KBr) *ν*_max_ 3425, 2927, 2858, 1632, 1384, 1031 cm^−1^; ^1^H NMR (600 MHz, CD_3_OD) data, see Table 1; ^13^C NMR (150 MHz, CD_3_OD) data, see Table 2; HRESIMS **1**: C_15_H_20_O_4_ *m/z* 287.1258 [M+Na]^+^ (calcd for C_15_H_20_O_4_Na, 287.1254); **2**: C_16_H_24_O_5_ *m/z* 319.1529 [M+Na]^+^ (calcd for C_16_H_24_O_5_Na, 319.1516).

#### 11,12-Epoxy-14-hydroxy-5,6-*seco*-tremula-1,6(13)-dien-5,12-olide (**3**) and methyl 12*β*,14-dihydroxy-5,6-*seco*-tremula-1,6(13)-dien-5-oate (**4**)

Colourless oil; $[\alpha ]_{{\text{D}}}^{{26}}$ − 77.5 (*c* 0.10, MeOH); UV (*c* 0.015 mg ml^−1^, MeOH) *λ*_max_ nm (Abs): 205 (0.4599), 238 (0.1222); IR (KBr) *ν*_max_ 3425, 2927, 2860, 1728, 1631, 1032 cm^−1^; ^1^H NMR (600 MHz, CD_3_OD) data, see Table 1; ^13^C NMR (150 MHz, CD_3_OD) data, see Table 2; HRESIMS **3**: C_15_H_20_O_4_ *m/z* 287.1250 [M+Na]^+^ (calcd for C_15_H_20_O_4_Na, 287.1254); **4**: C_16_H_24_O_5_ *m/z* 319.1513 [M+Na]^+^ (calcd for C_16_H_24_O_5_Na, 319.1516).

#### 6,11-Dihydroxy-5,6-*seco*-tremul-1-en-5,12-olide (**5**)

Colourless oil; C_15_H_24_O_4_; $[\alpha ]_{{\text{D}}}^{{26}}$ + 14.6 (*c* 0.07, MeOH); UV (MeOH) *λ*_max_ nm (log *ε*): 206 (4.13), 254 (2.97); IR (KBr) *ν*_max_ 3422, 2952, 2930, 2868, 1750, 1632, 1383, 1193, 1022 cm^−1^; ^1^H NMR (600 MHz, CD_3_OD) data, see Table 1; ^13^C NMR (150 MHz, CD_3_OD) data, see Table 2; HRESIMS *m/z* 291.1572 [M +Na]^+^ (calcd for C_15_H_24_O_4_Na, 291.1567).

#### 11,12-Epoxy-6-hydroxy-5,6-*seco*-tremul-1-en-5,12-olide (**6**)

Colourless oil; C_15_H_22_O_4_; $[\alpha ]_{{\text{D}}}^{{26}}$ + 47.8 (*c* 0.07, MeOH); UV (MeOH) *λ*_max_ nm (log *ε*): 205 (3.46), 245 (2.18); IR (KBr) *ν*_max_ 3432, 2927, 2859, 1631, 1385, 1031 cm^−1^; ^1^H NMR (800 MHz, CDCl_3_) data, see Table 1; ^13^C NMR (200 MHz, CDCl_3_) data, see Table 2; HRESIMS *m/z* 289.1416 [M+Na]^+^ (calcd for C_15_H_22_O_4_Na, 289.1410).

## Electronic supplementary material

Below is the link to the electronic supplementary material.

Supplementary material 1 (DOCX 7398 kb)
