# Supplementary material for: Seco-tremulane Sesquiterpenoids from the Cultures of the Medicinal Fungus Irpex lacteus HFG1102
Source: Nat Prod Bioprospect. 2018 Mar 19;8(2):113–9. doi: 10.1007/s13659-018-0157-y (PMC5913050; doi:10.1007/s13659-018-0157-y)

**Supplementary data for**

***Seco*-tremulane Sesquiterpenoids from the Cultures of the Medicinal Fungus *Irpex lacteus* HFG1102**

He-Ping Chen, Zhen-Zhu Zhao, Zheng-Hui Li, Tao Feng,* Ji-Kai Liu*

*School of Pharmaceutical Sciences, South-Central University for Nationalities, Wuhan 430074, People’s Republic of China*

**Corresponding authors**

*E-mail: [tfeng@mail.scuec.edu.cn](mailto:tfeng@mail.scuec.edu.cn) (T. Feng)

*E-mail: [jkliu@mail.kib.ac.cn](mailto:jkliu@mail.kib.ac.cn) (J.-K. Liu)

**Contents**

[Figure 1S. ^1^H NMR spectrum of **1/2** (600 MHz, CD_3_OD). S2](#_Toc504527630)

[Figure 2S. ^13^C NMR and DEPT spectra of **1/2** (150 MHz, CD_3_OD). S3](#_Toc504527631)

[Figure 3S. HSQC spectrum of **1/2**. S4](#_Toc504527632)

[Figure 4S. ^1^H-^1^H COSY spectrum of **1/2**. S5](#_Toc504527633)

[Figure 5S. HMBC spectrum of **1/2**. S6](#_Toc504527634)

[Figure 6S. ROESY spectrum of **1/2**. S7](#_Toc504527635)

[Figure 7S. (+)-HRESIMS report of **1**. S8](#_Toc504527636)

[Figure 8S. (+)-HRESIMS report of **2**. S9](#_Toc504527637)

[Figure 9S. ^1^H NMR spectrum of **3/4** (600 MHz, CD_3_OD). S10](#_Toc504527638)

[Figure 10S. ^13^C NMR and DEPT spectra of **3/4** (150 MHz, CD_3_OD). S11](#_Toc504527639)

[Figure 11S. HSQC spectrum of **3/4**. S12](#_Toc504527640)

[Figure 12S. ^1^H-^1^H COSY spectrum of **3/4**. S13](#_Toc504527641)

[Figure 13S. HMBC spectrum of **3/4**. S14](#_Toc504527642)

[Figure 14S. ROESY spectrum of **3/4**. S15](#_Toc504527643)

[Figure 15S. (+)-HRESIMS report of **3**. S16](#_Toc504527644)

[Figure 16S. (+)-HRESIMS report of **4**. S17](#_Toc504527645)

[Figure 17S. ^1^H NMR spectrum of **5** (600 MHz, CD_3_OD). S18](#_Toc504527646)

[Figure 18S. ^13^C NMR and DEPT spectra of **5** (150 MHz, CD_3_OD). S19](#_Toc504527647)

[Figure 19S. HSQC spectrum of **5**. S20](#_Toc504527648)

[Figure 20S. ^1^H-^1^H COSY spectrum of **5**. S21](#_Toc504527649)

[Figure 21S. HMBC spectrum of **5**. S22](#_Toc504527650)

[Figure 22S. ROESY spectrum of **5**. S23](#_Toc504527651)

[Figure 23S. (+)-HRESIMS report of **5**. S24](#_Toc504527652)

[Figure 24S. ^1^H NMR spectrum of **6** (800 MHz, CDCl_3_). S25](#_Toc504527653)

[Figure 25S. ^13^C NMR spectrum of **6** (200 MHz, CDCl_3_). S26](#_Toc504527654)

[Figure 26S. HSQC spectrum of **6**. S27](#_Toc504527655)

[Figure 27S. ^1^H-^1^H COSY spectrum of **6**. S28](#_Toc504527656)

[Figure 28S. HMBC spectrum of **6**. S29](#_Toc504527657)

[Figure 29S. ROESY spectrum of **6**. S30](#_Toc504527658)

[Figure 30S. (+)-HRESIMS report of **6**. S31](#_Toc504527659)

# Figure 1S. ^1^H NMR spectrum of **1/2** (600 MHz, CD_3_OD).


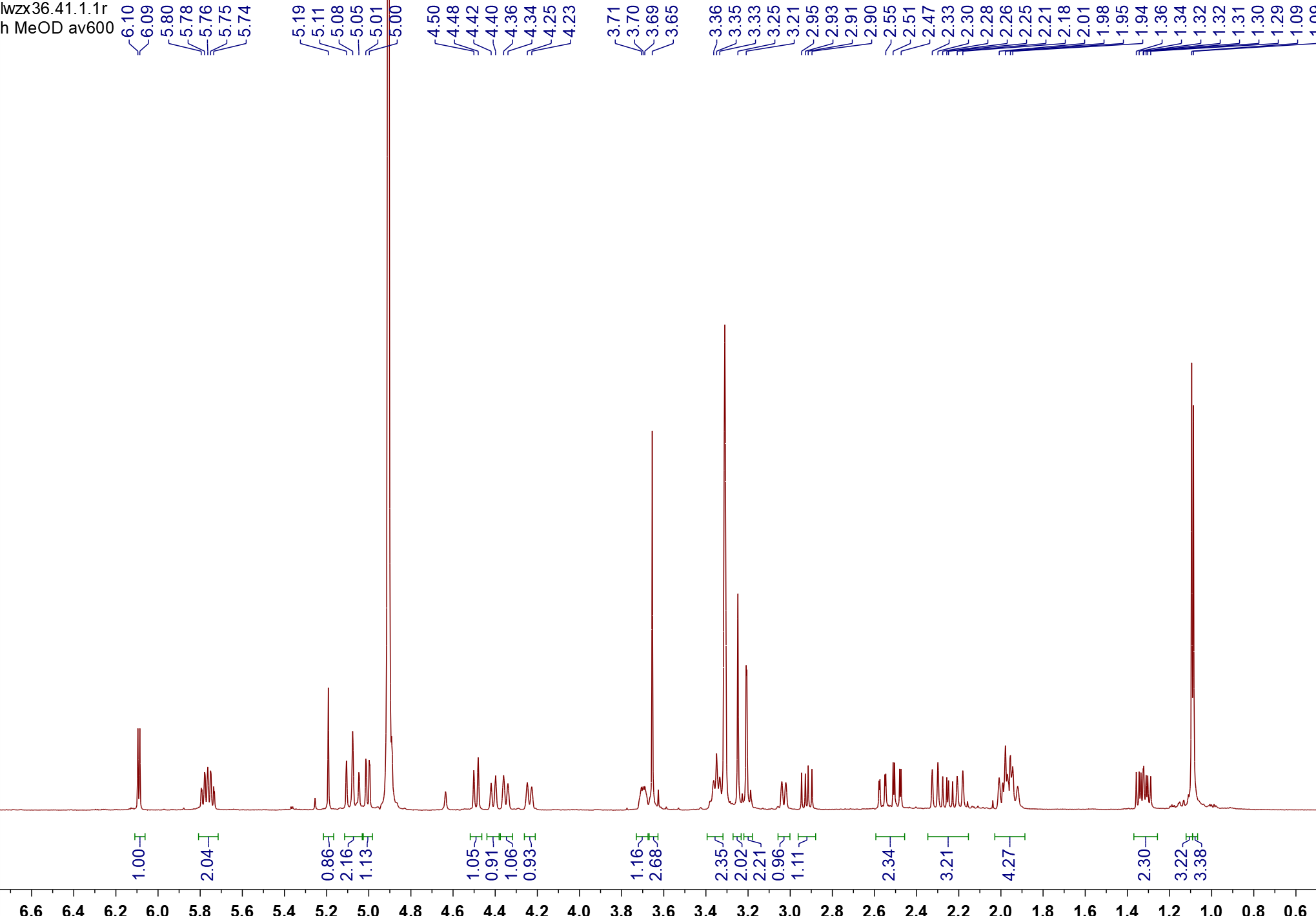


# Figure 2S. ^13^C NMR and DEPT spectra of **1/2** (150 MHz, CD_3_OD).


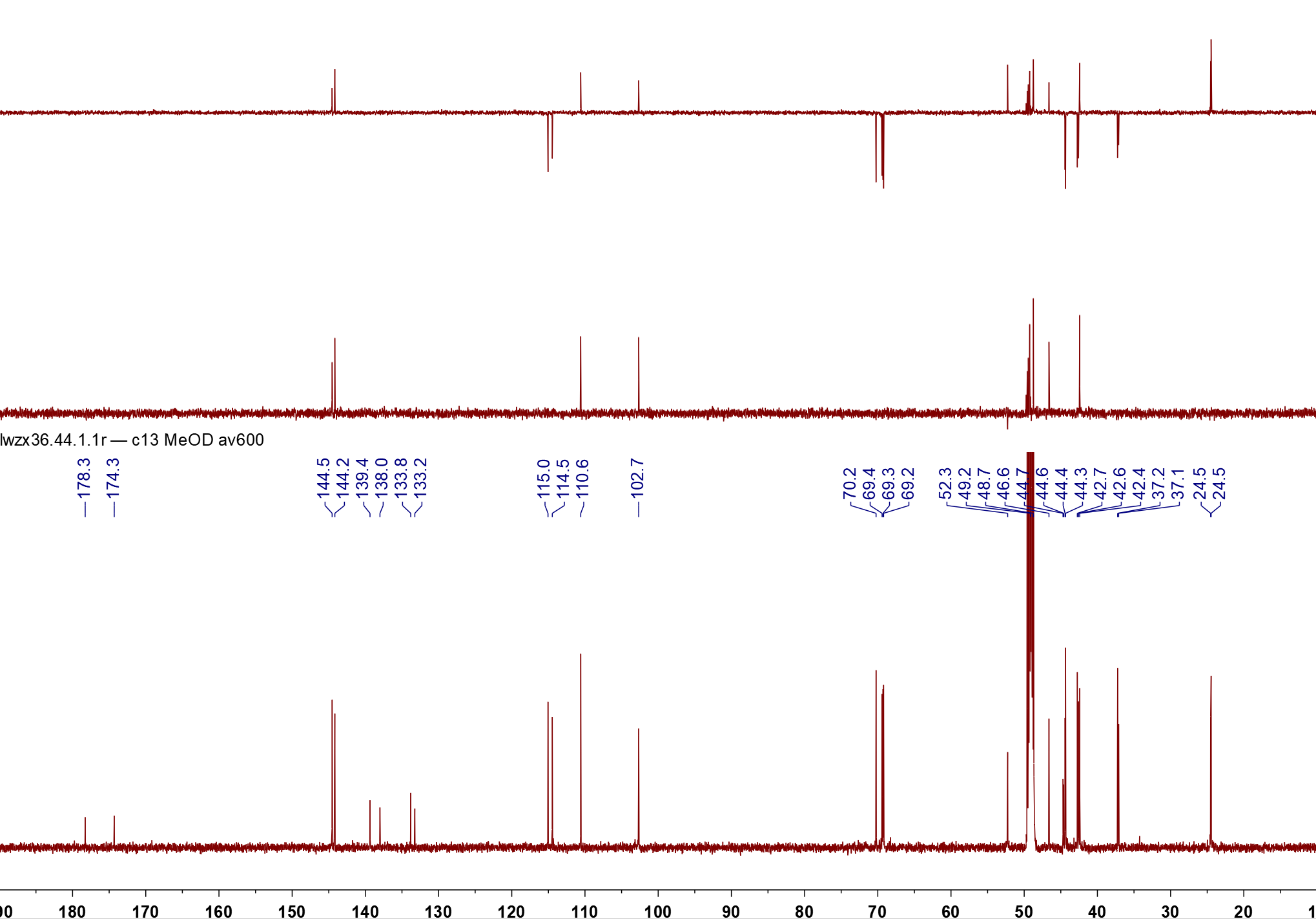


# Figure 3S. HSQC spectrum of **1/2**.


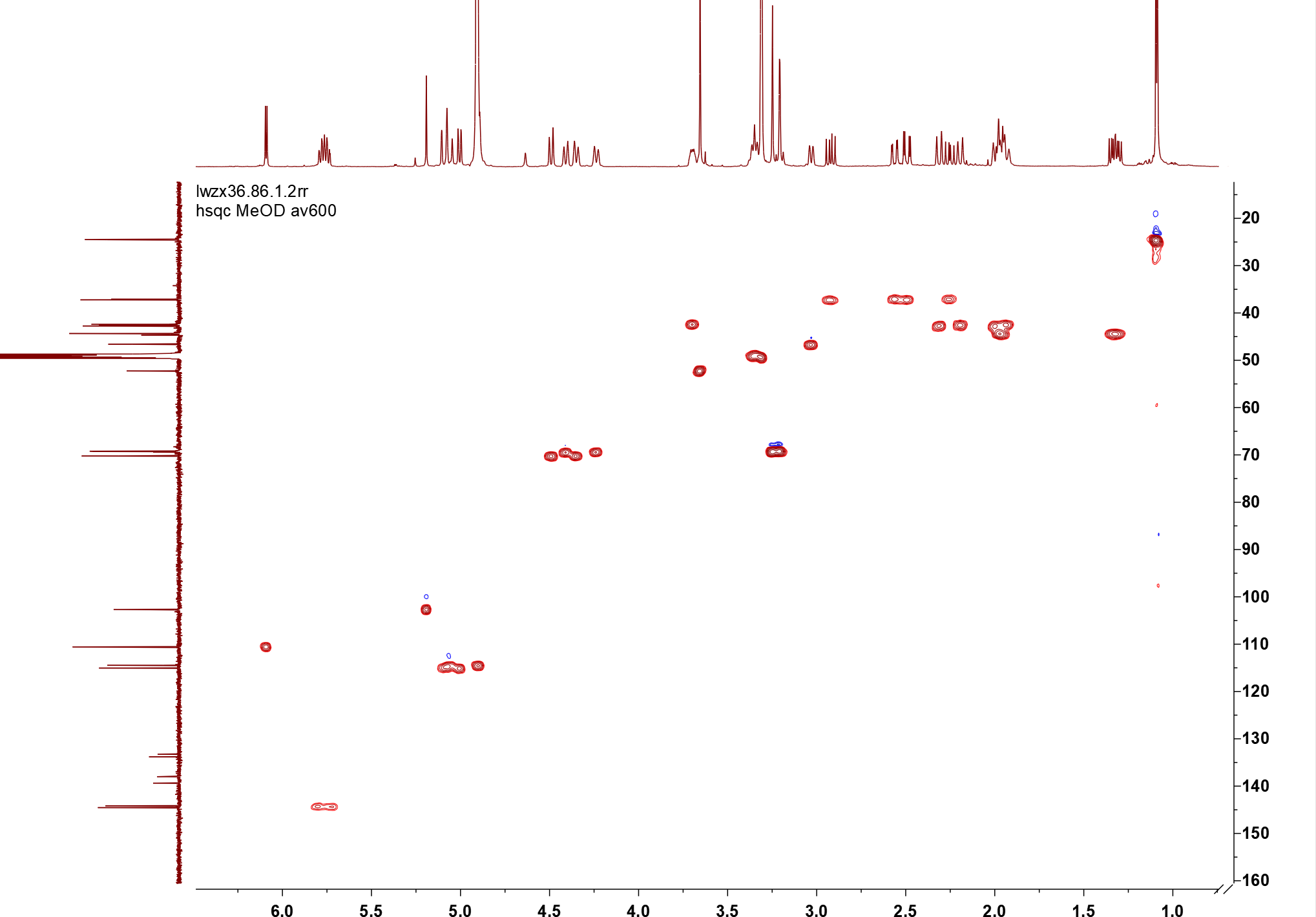


# Figure 4S. ^1^H-^1^H COSY spectrum of **1/2**.


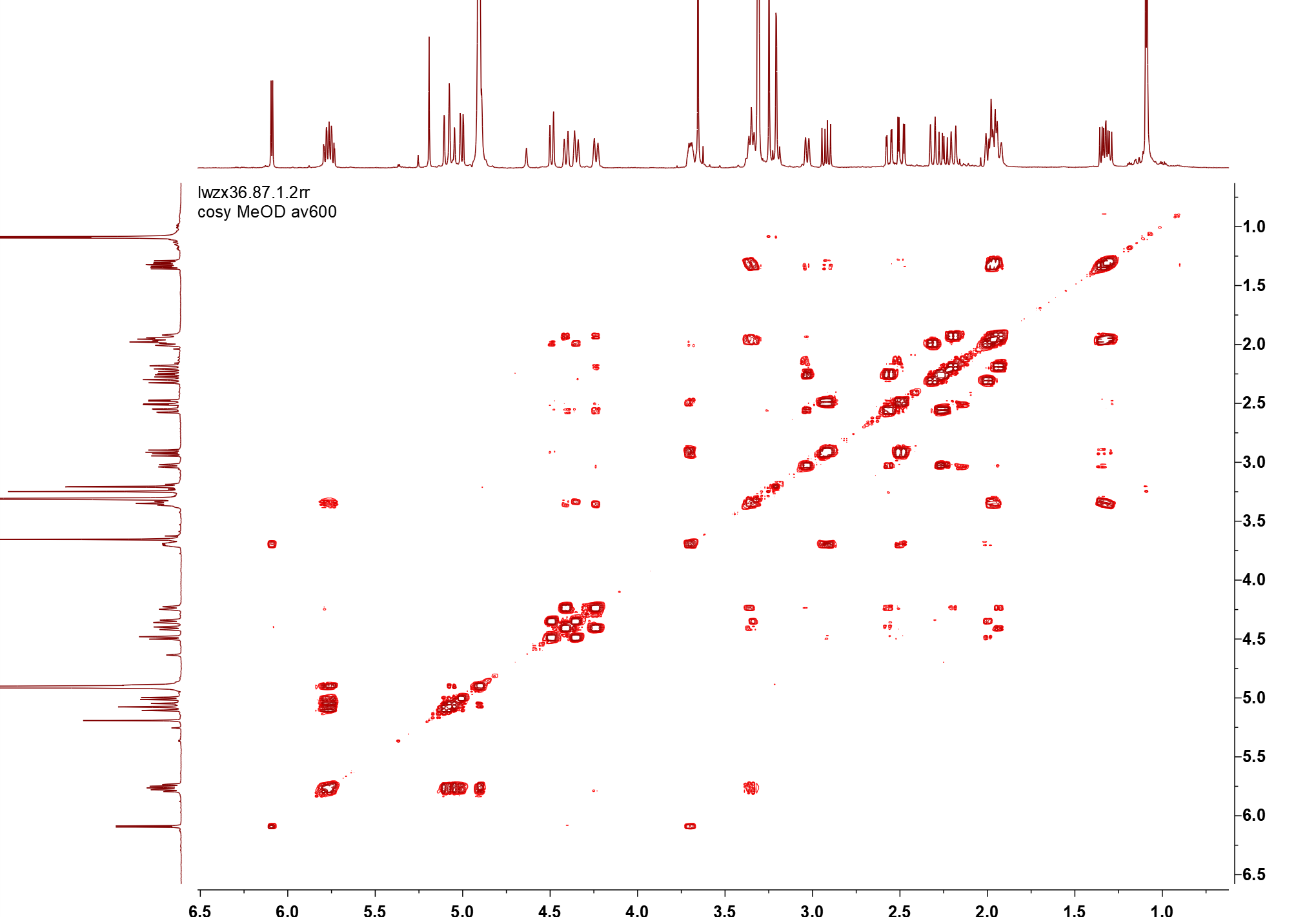


# Figure 5S. HMBC spectrum of **1/2**.


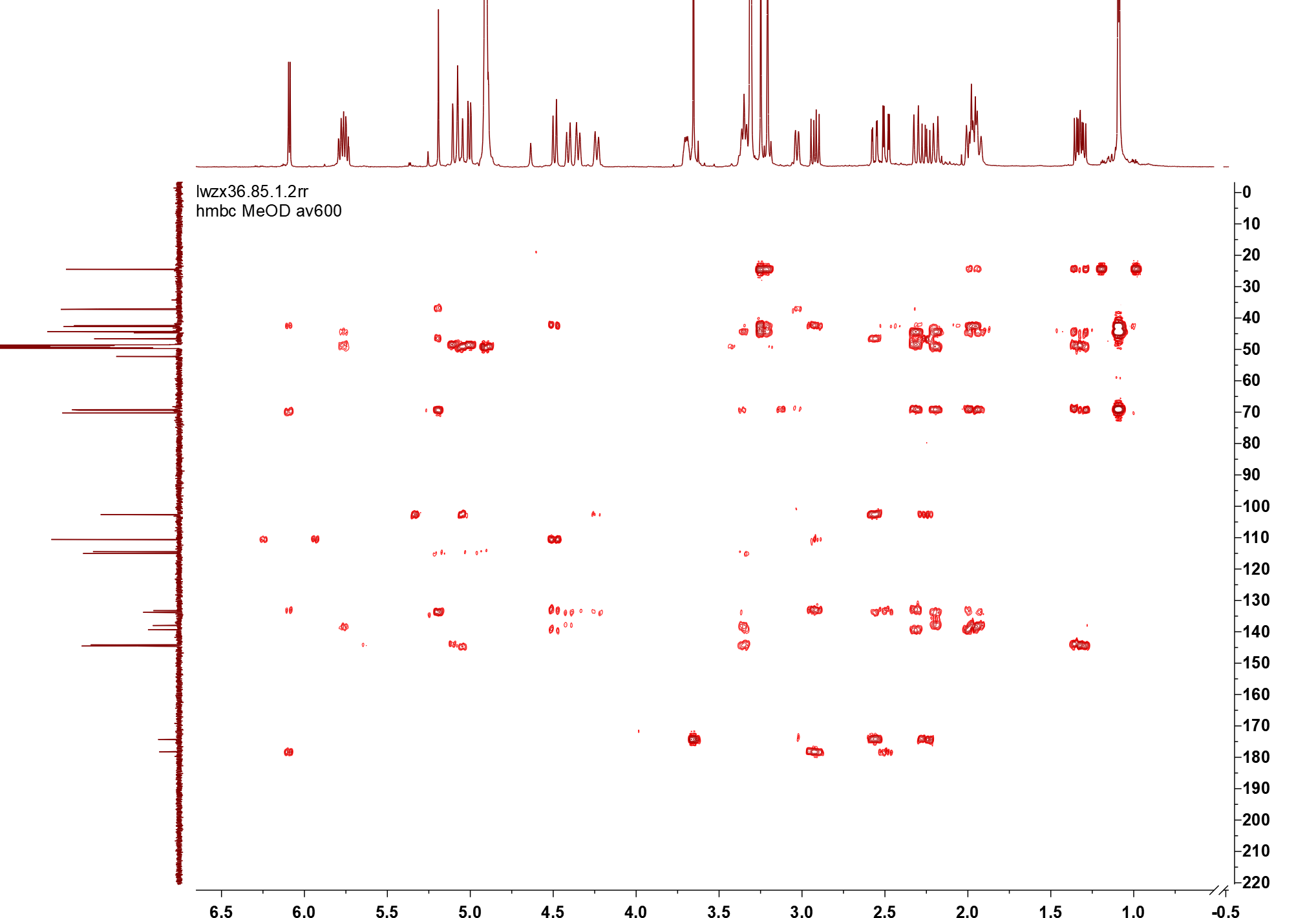


# Figure 6S. ROESY spectrum of **1/2**.


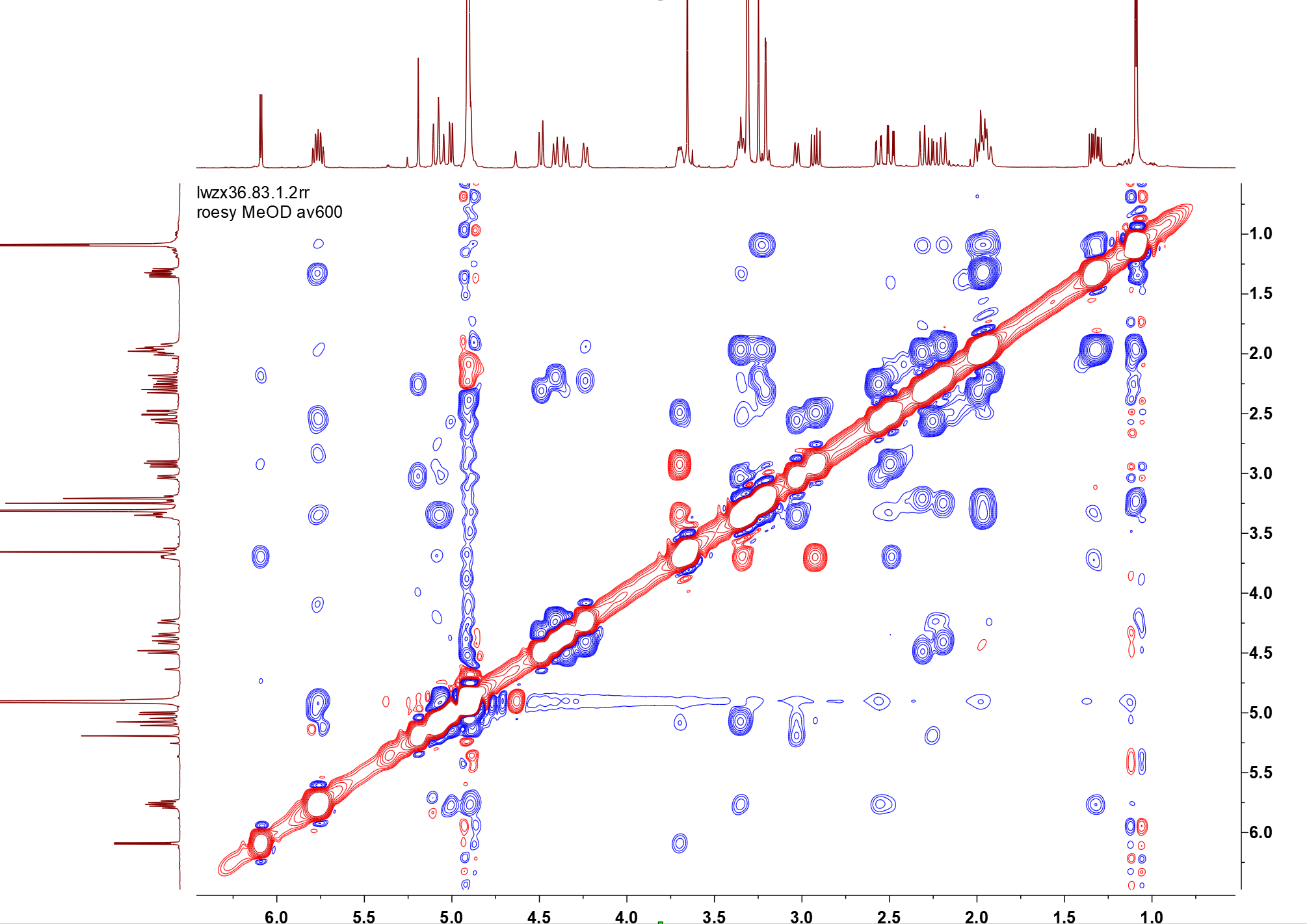


# Figure 7S. (+)-HRESIMS report of **1**.


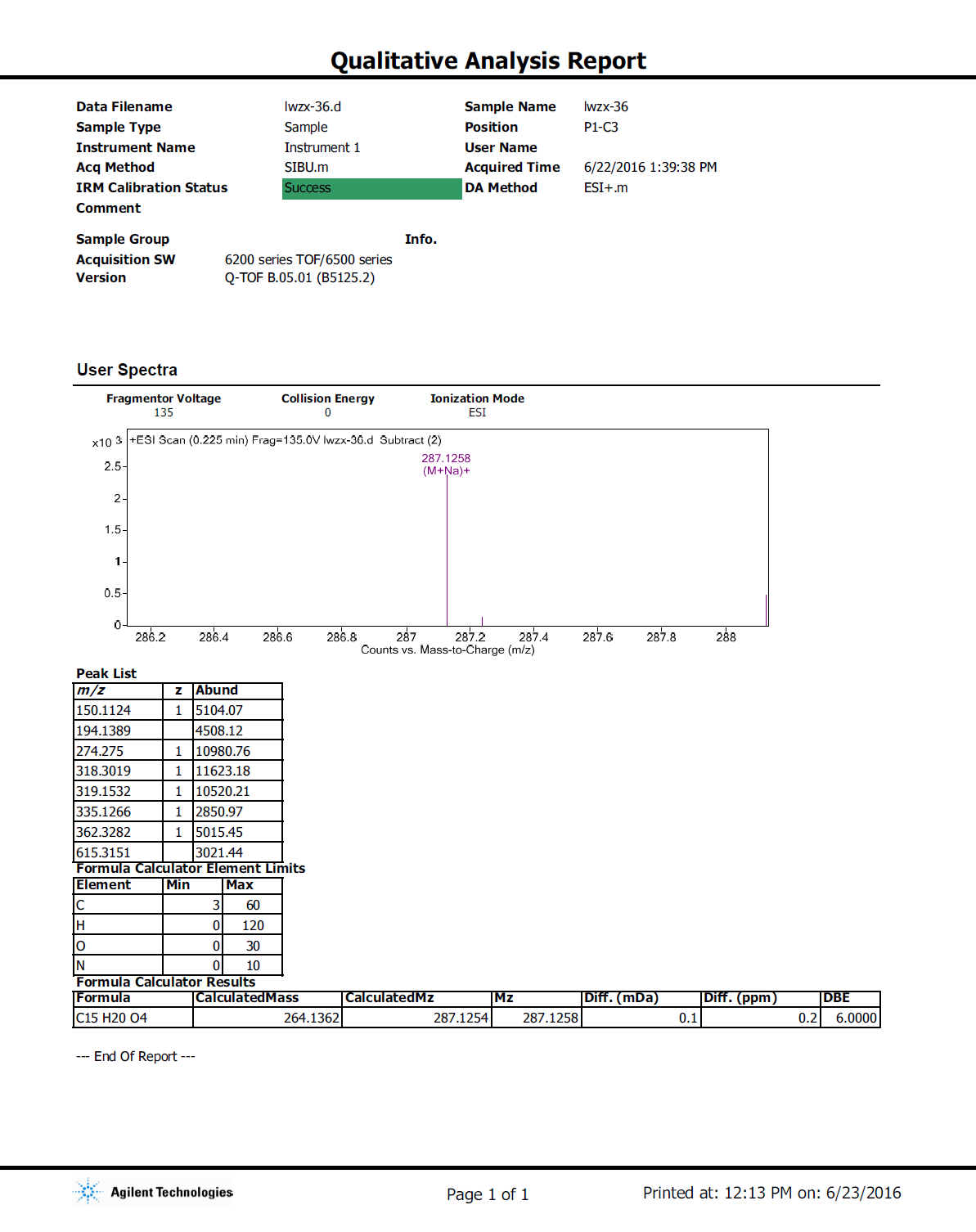


# Figure 8S. (+)-HRESIMS report of **2**.


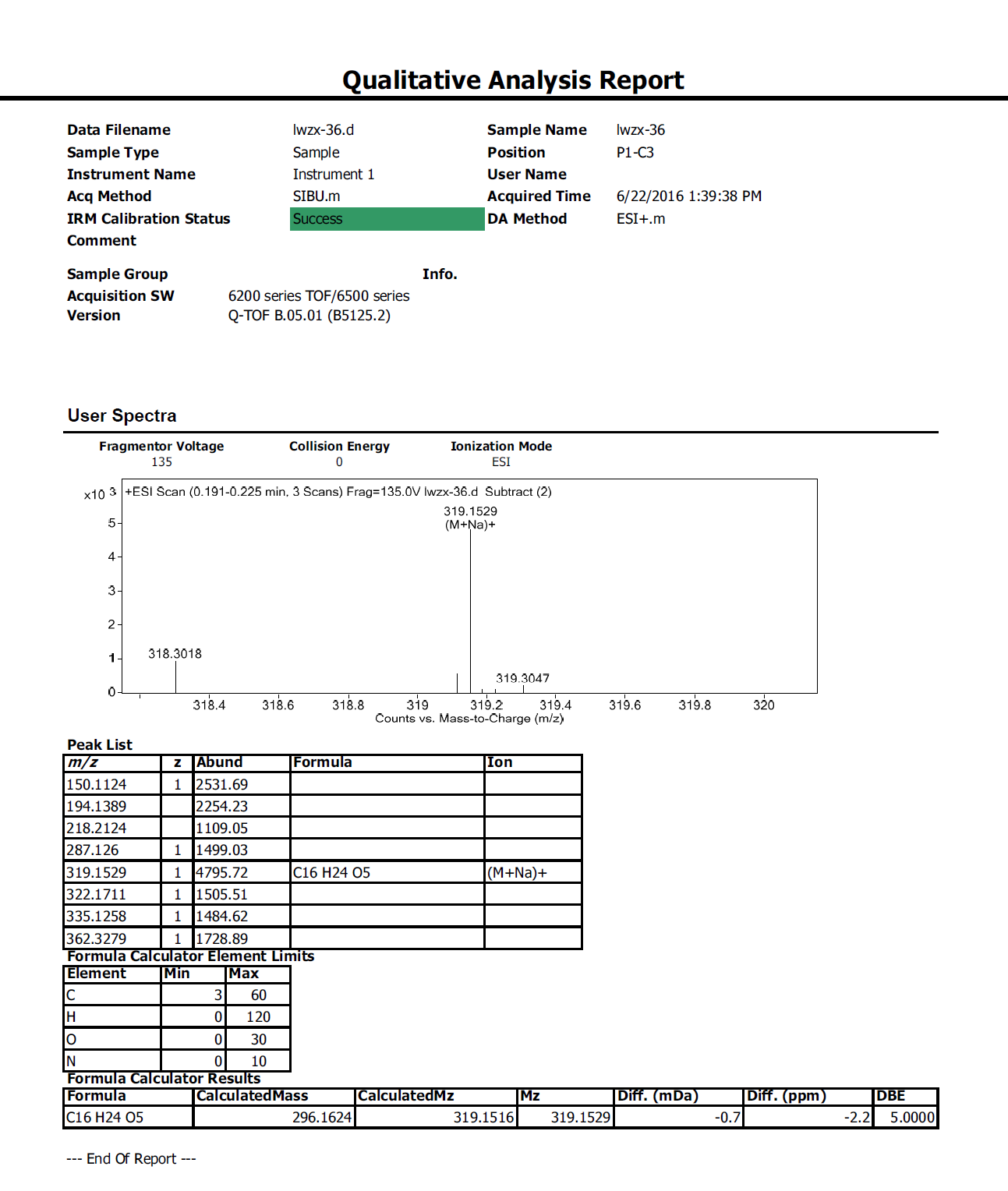


# Figure 9S. ^1^H NMR spectrum of **3/4** (600 MHz, CD_3_OD).


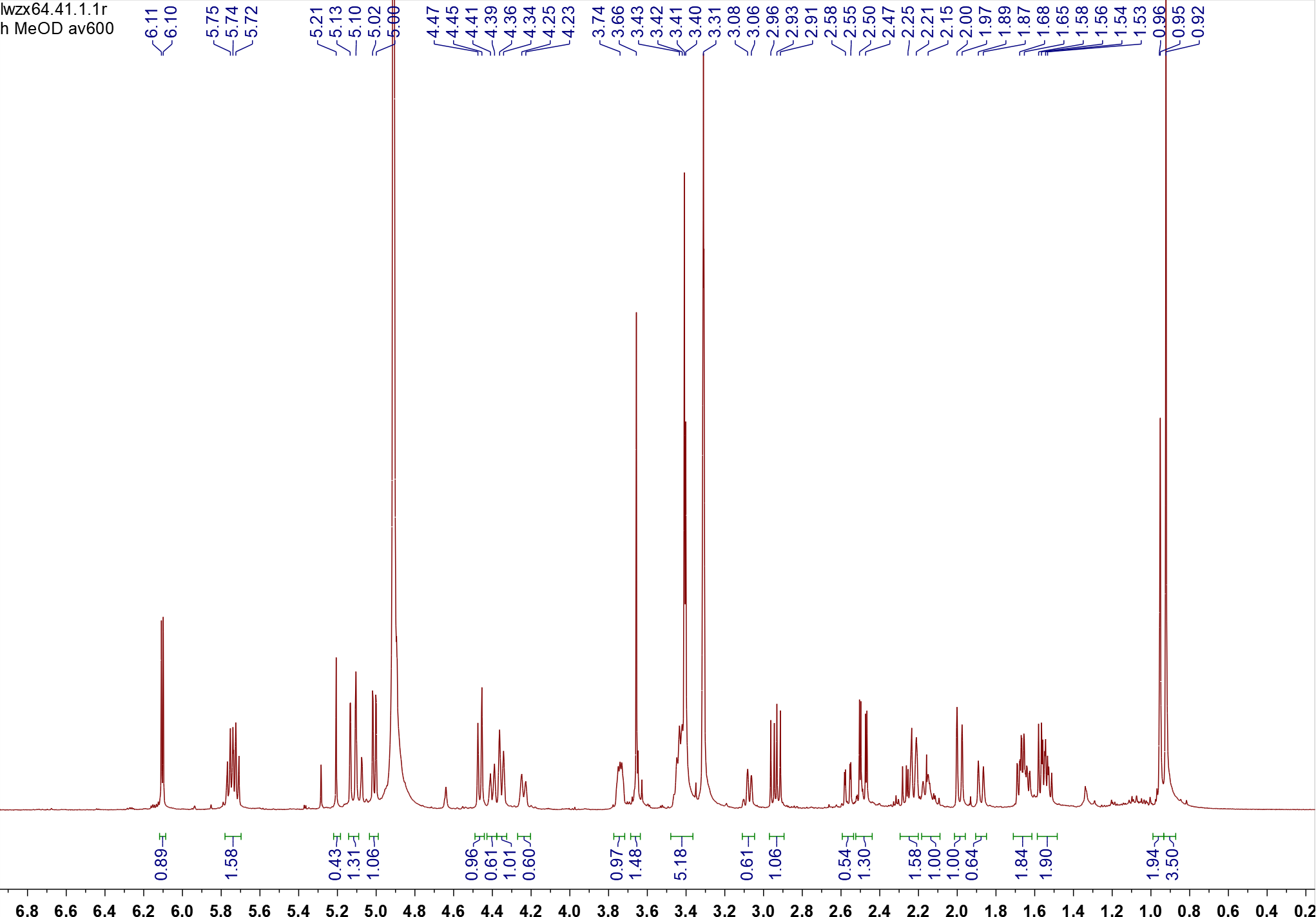


# Figure 10S. ^13^C NMR and DEPT spectra of **3/4** (150 MHz, CD_3_OD).


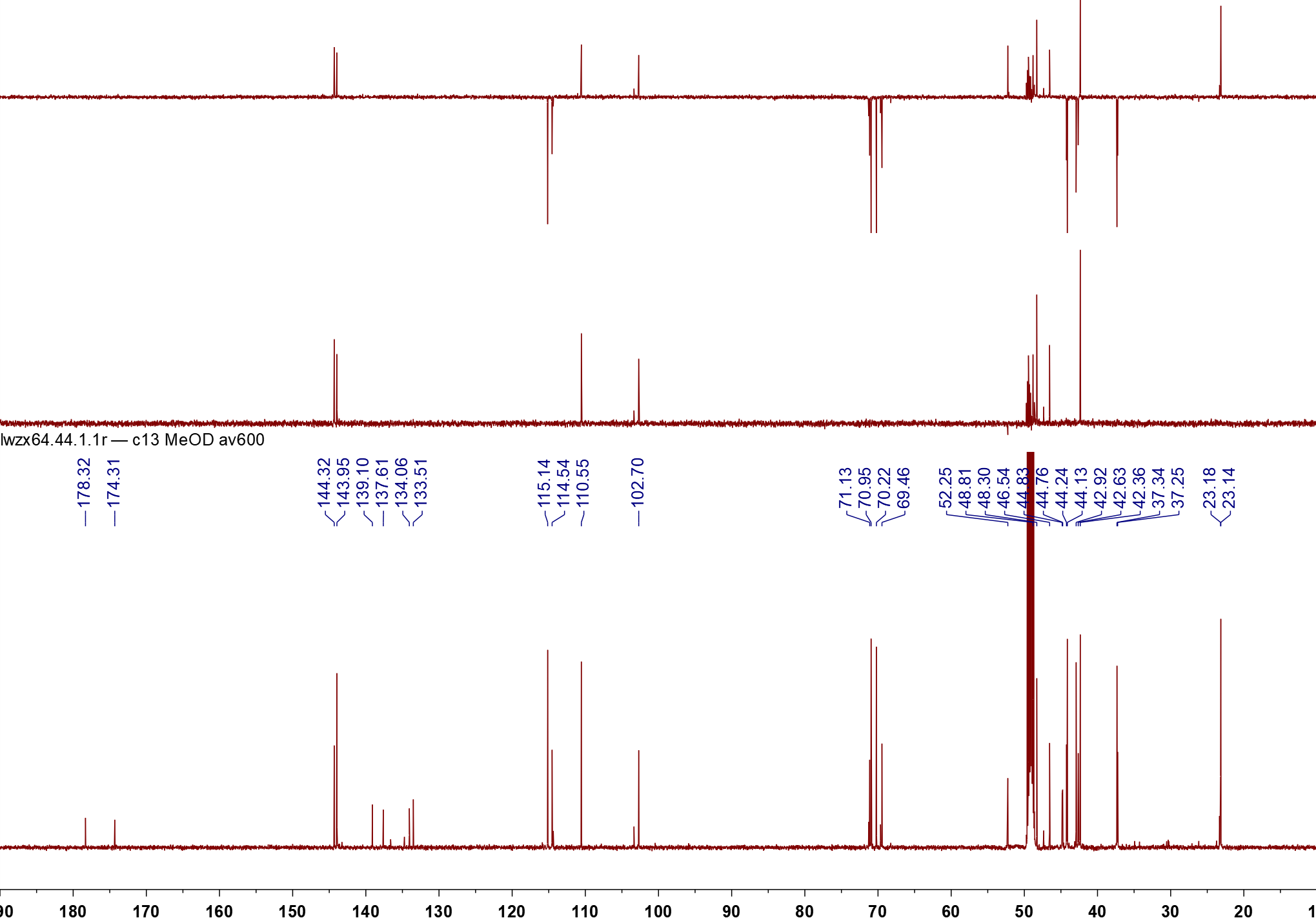


# Figure 11S. HSQC spectrum of **3/4**.


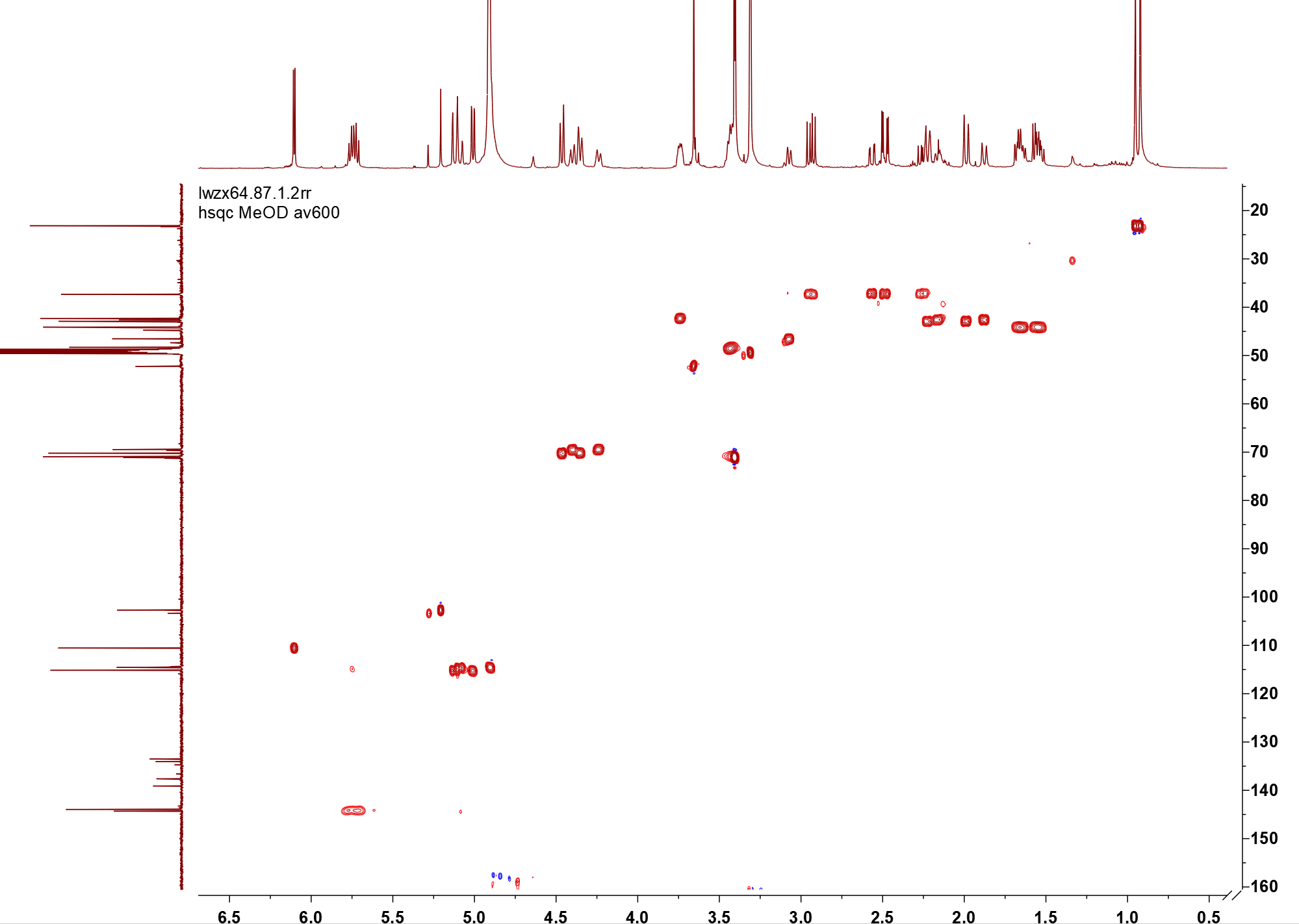


# Figure 12S. ^1^H-^1^H COSY spectrum of **3/4**.


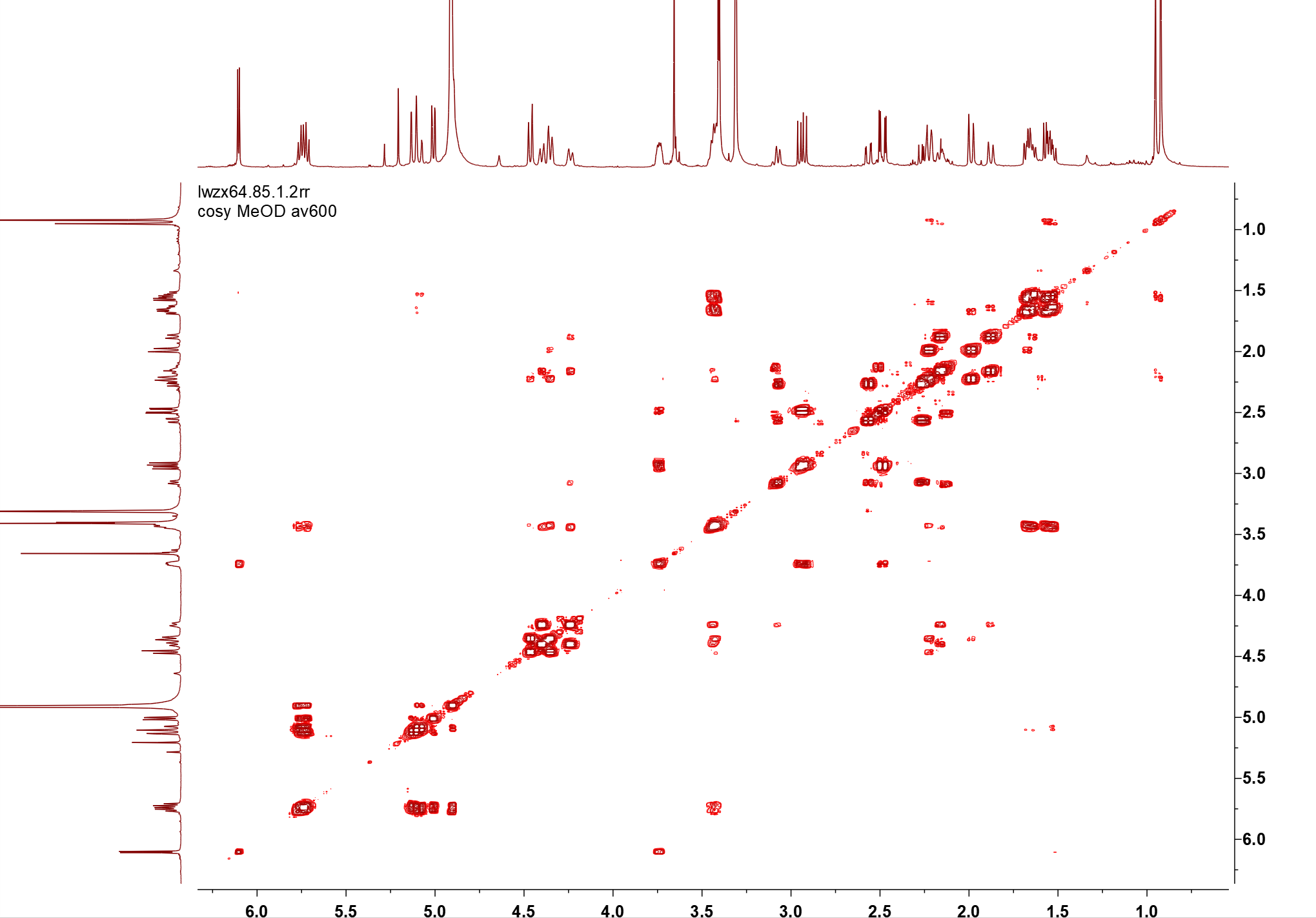


# Figure 13S. HMBC spectrum of **3/4**.


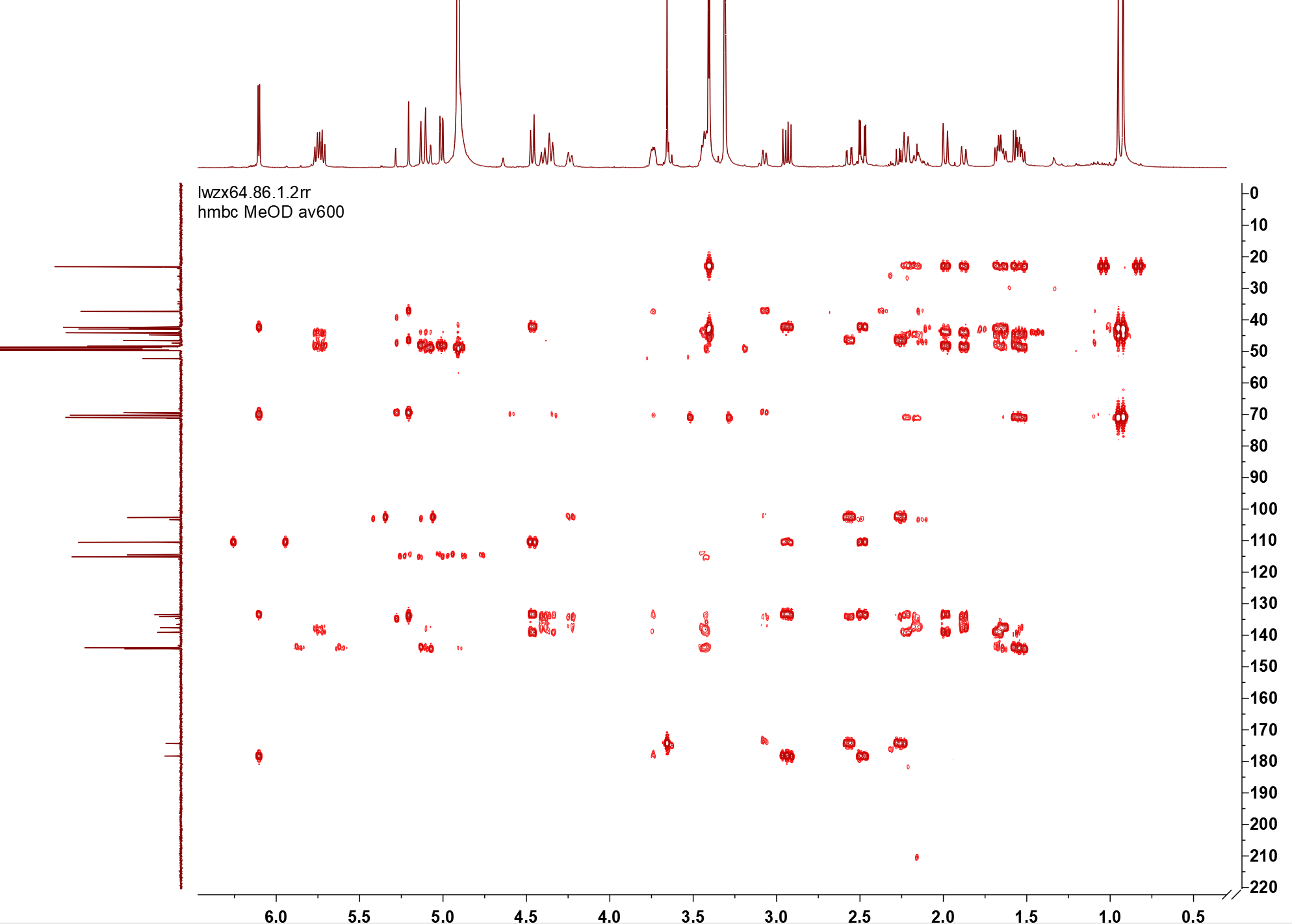


# Figure 14S. ROESY spectrum of **3/4**.


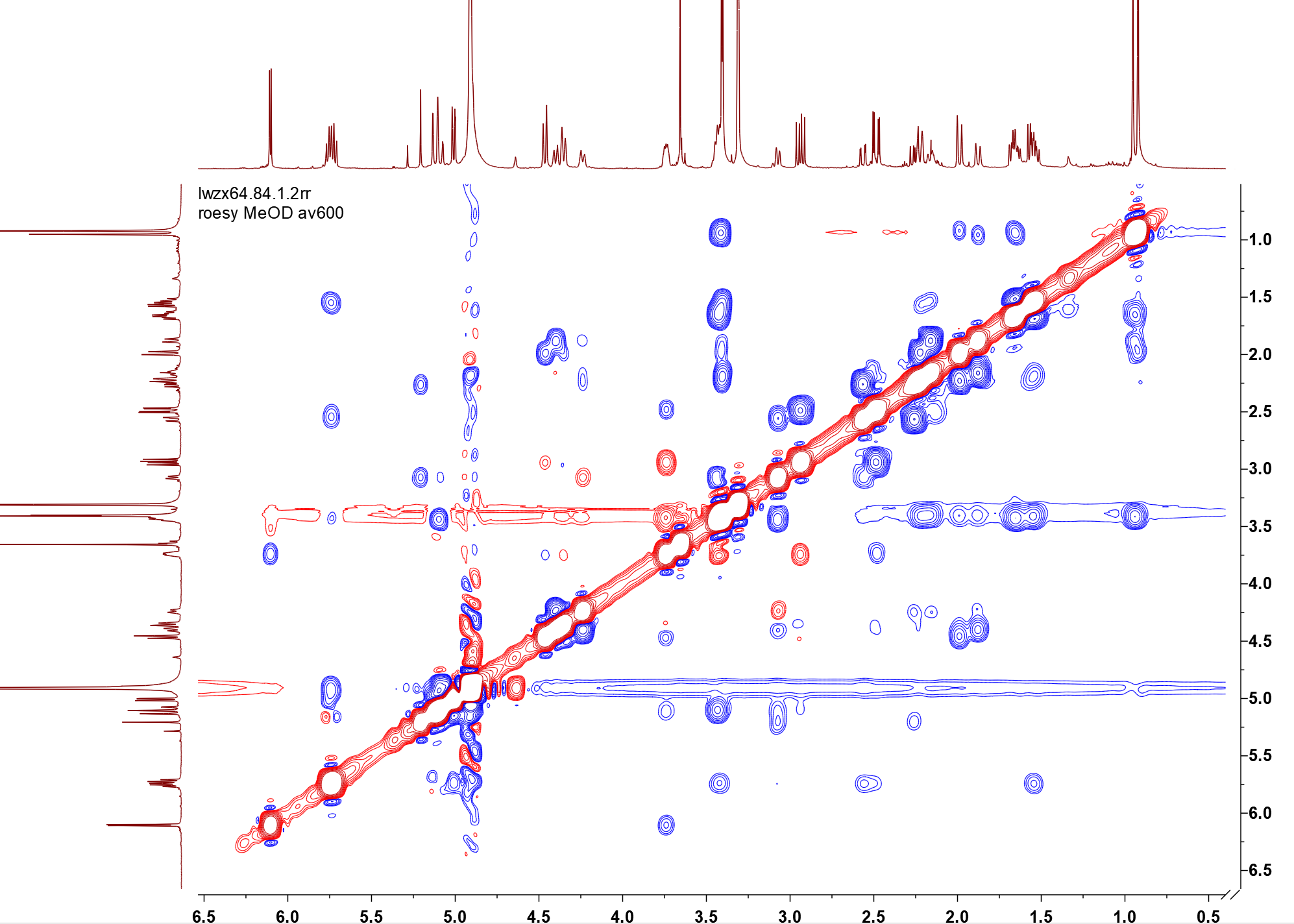


# Figure 15S. (+)-HRESIMS report of **3**.


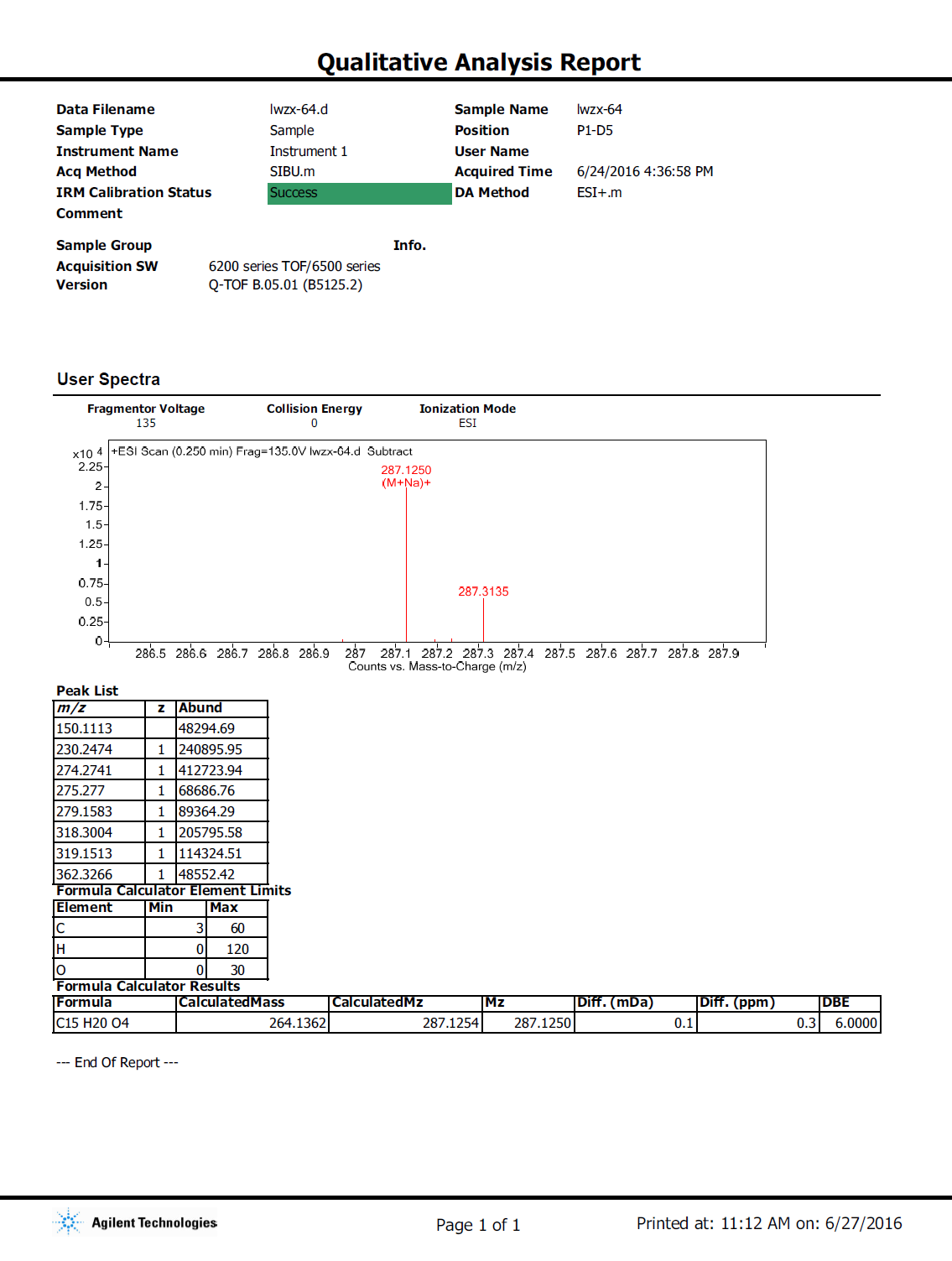


# Figure 16S. (+)-HRESIMS report of **4**.


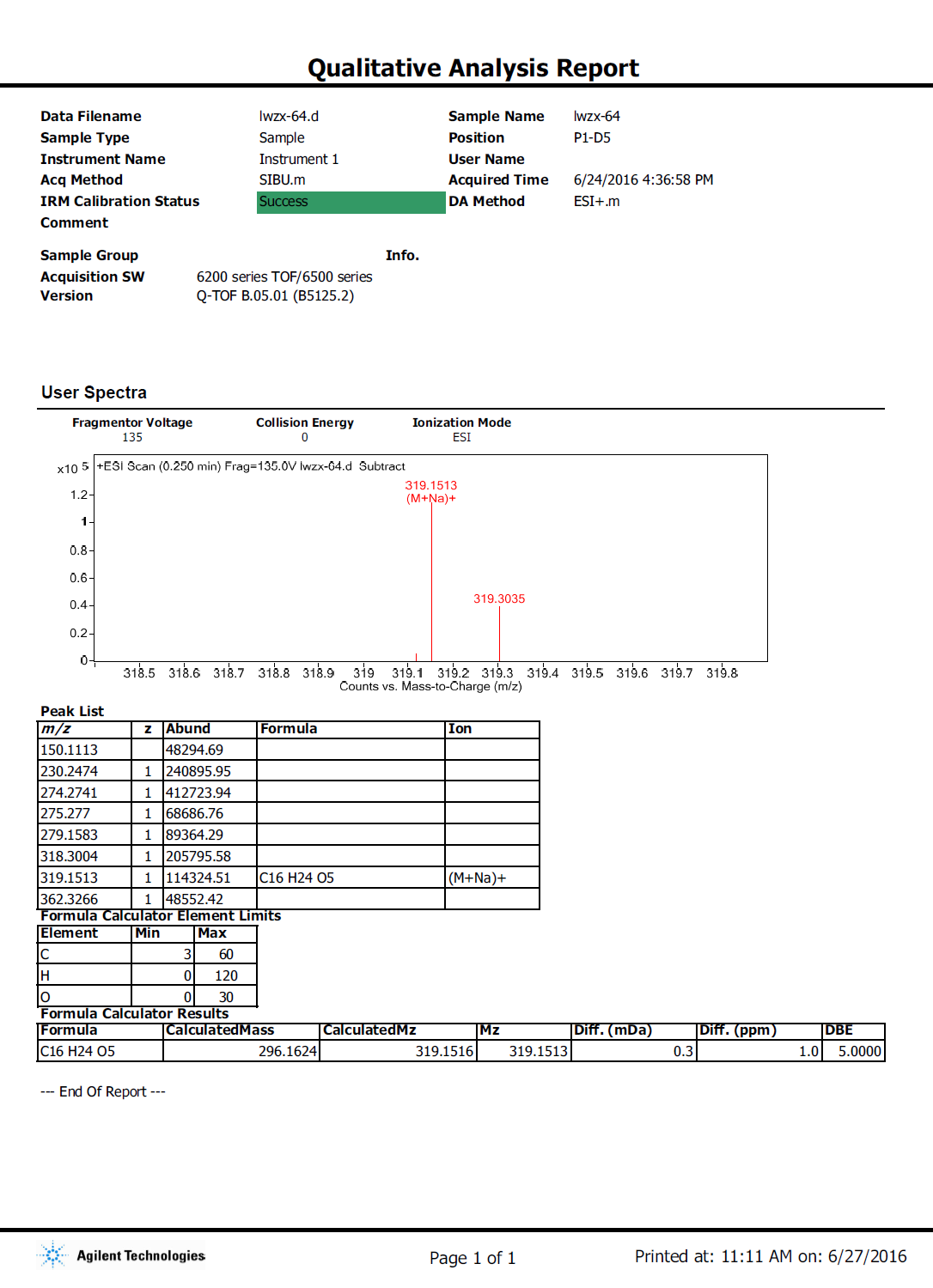


# Figure 17S. ^1^H NMR spectrum of **5** (600 MHz, CD_3_OD).


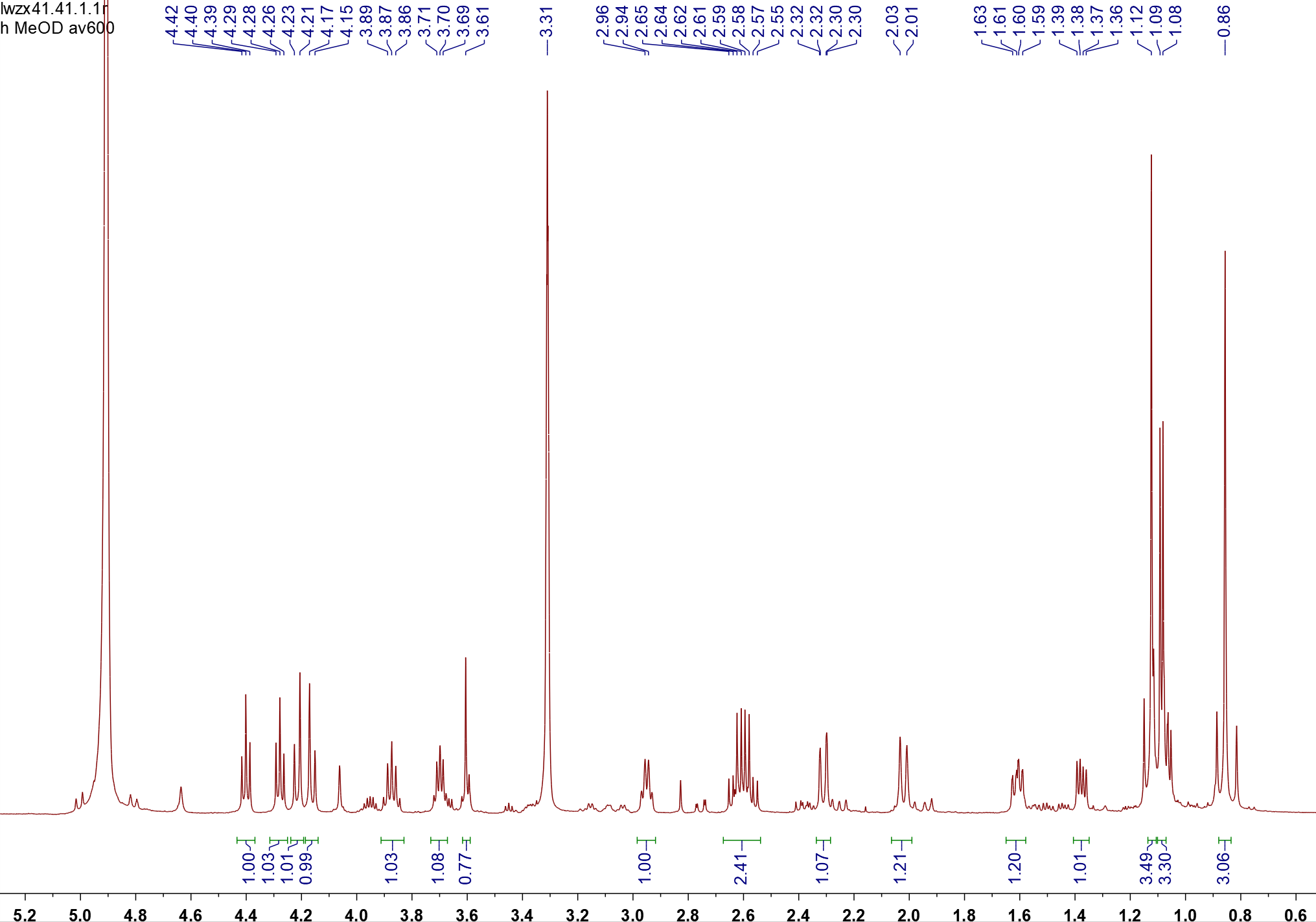


# Figure 18S. ^13^C NMR and DEPT spectra of **5** (150 MHz, CD_3_OD).


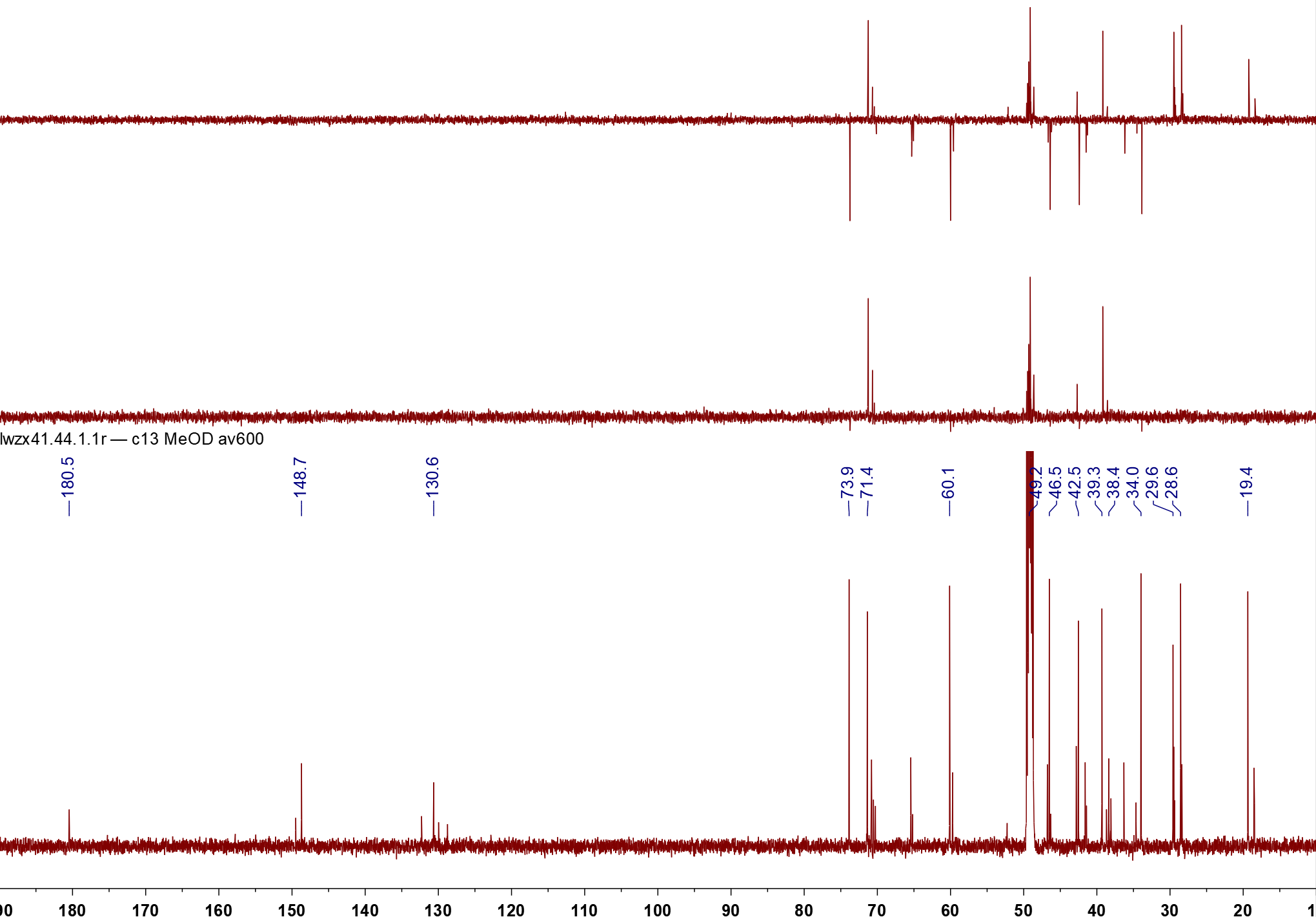


# Figure 19S. HSQC spectrum of **5**.


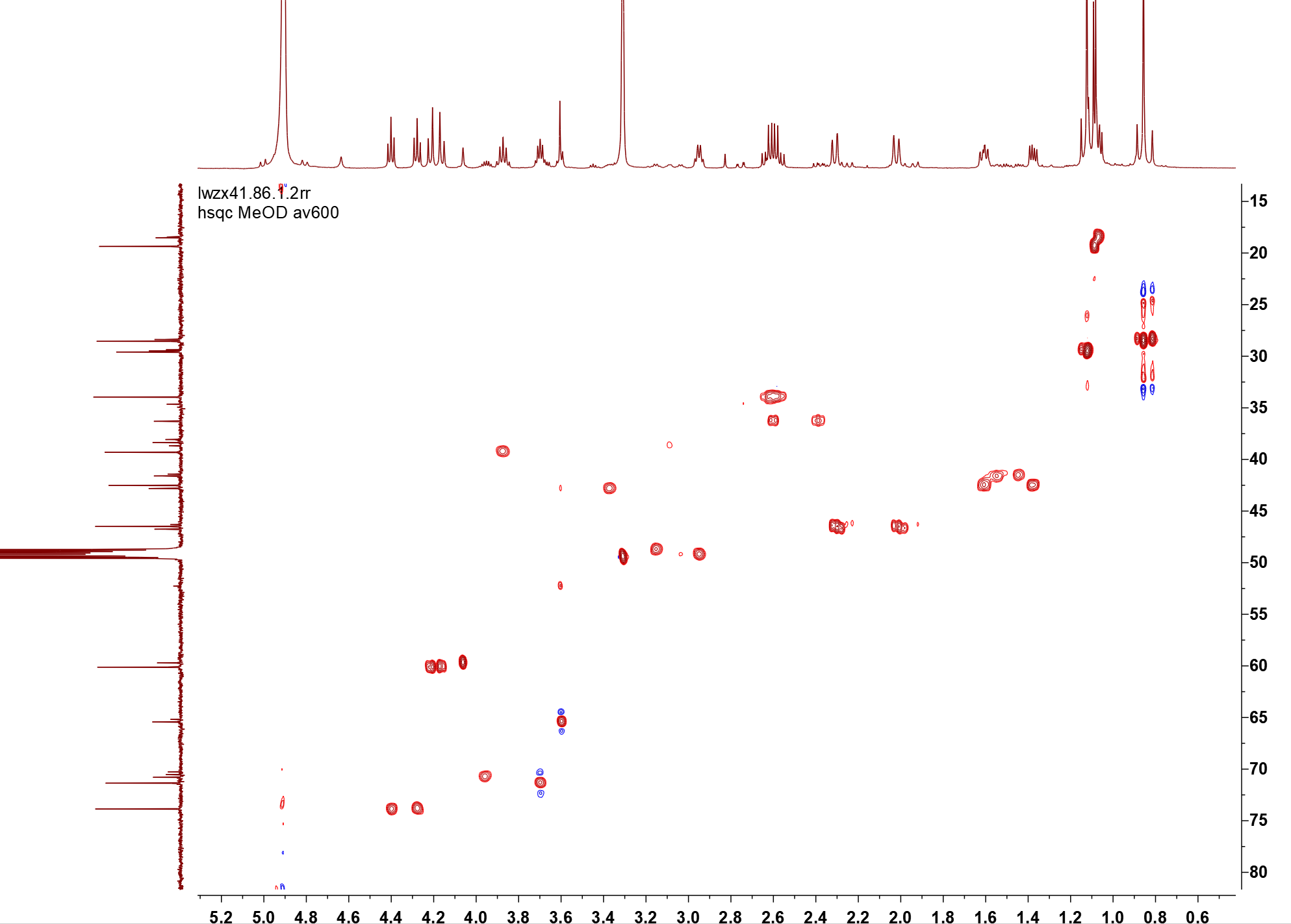


# Figure 20S. ^1^H-^1^H COSY spectrum of **5**.


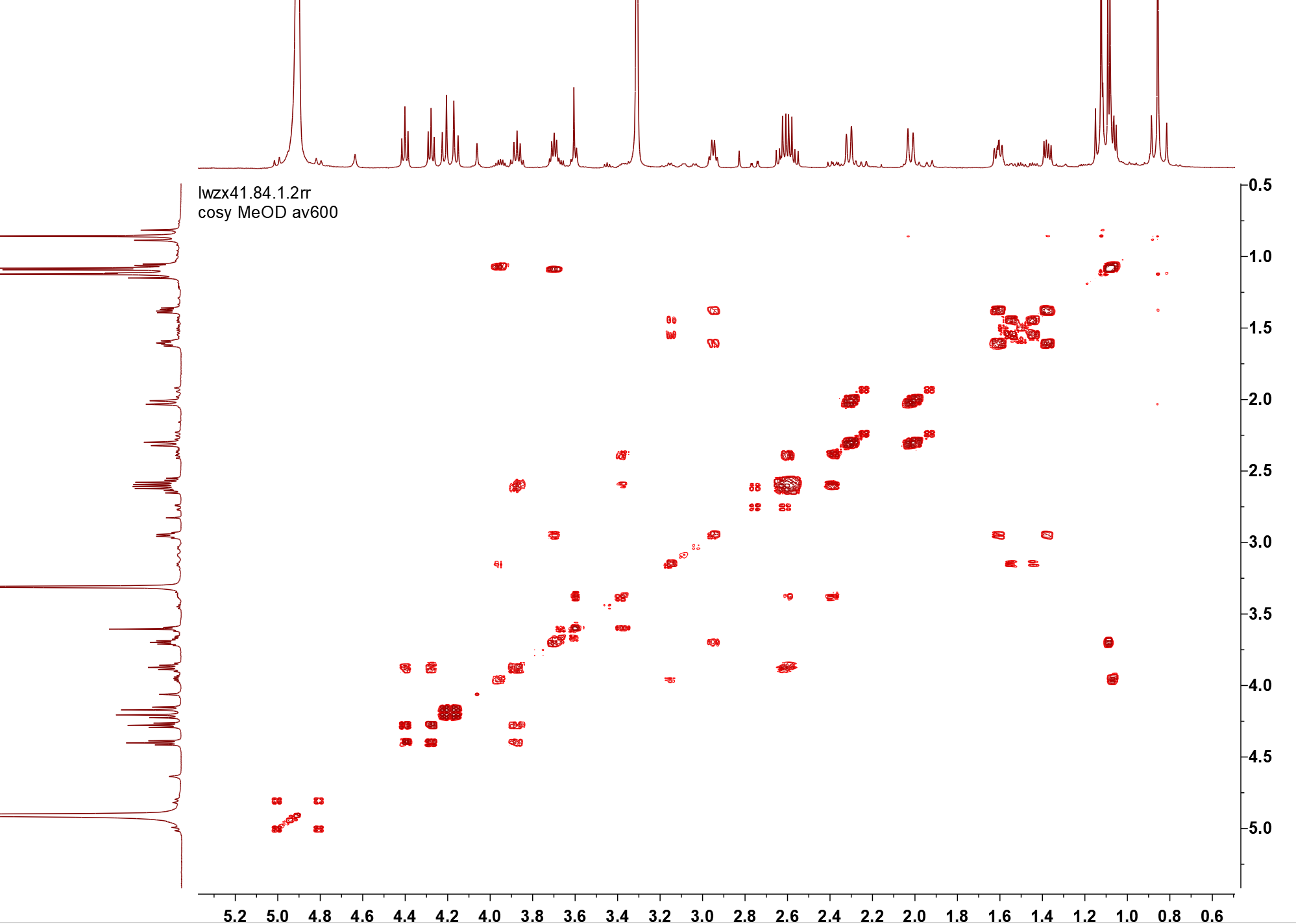


# Figure 21S. HMBC spectrum of **5**.


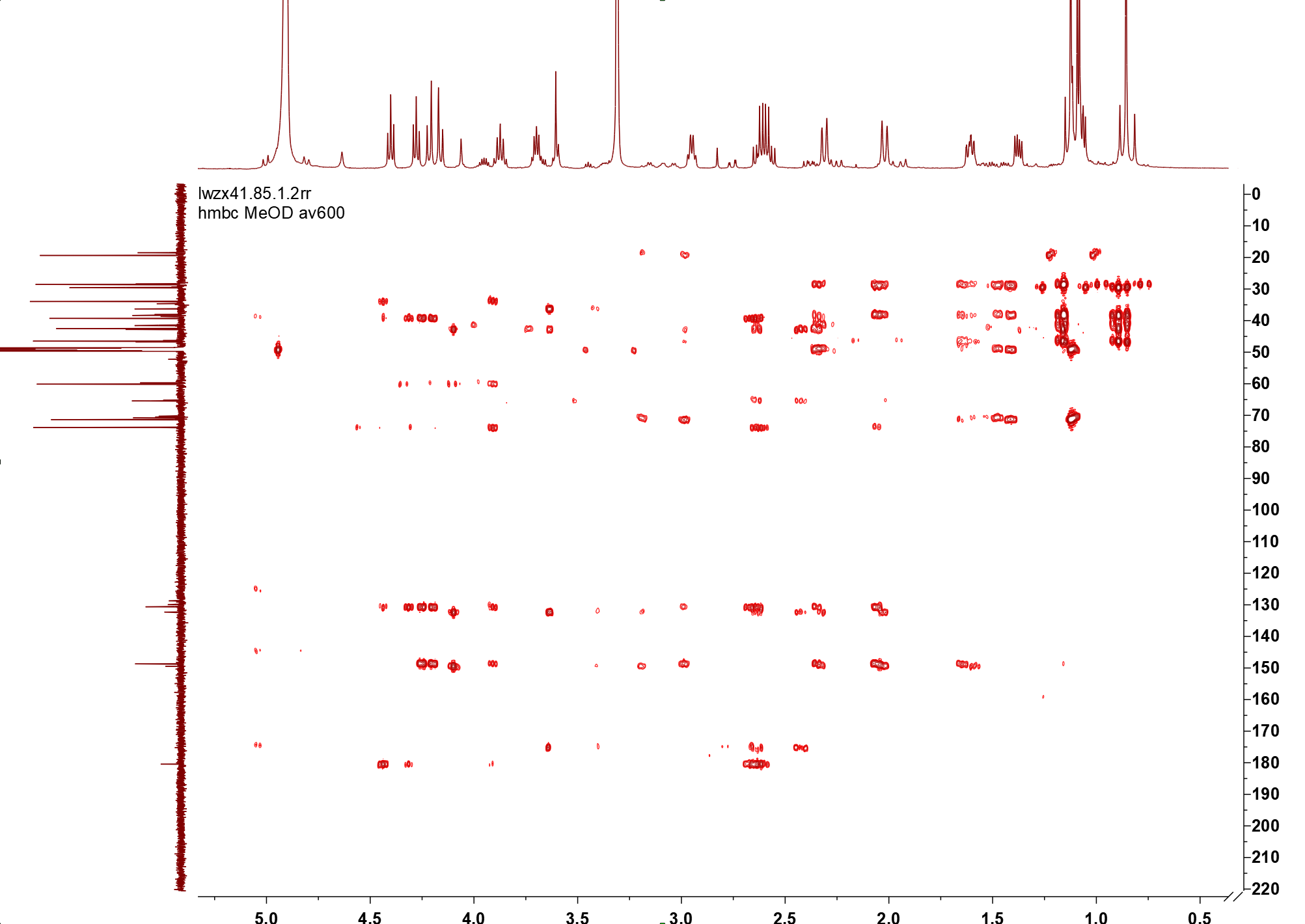


# Figure 22S. ROESY spectrum of **5**.


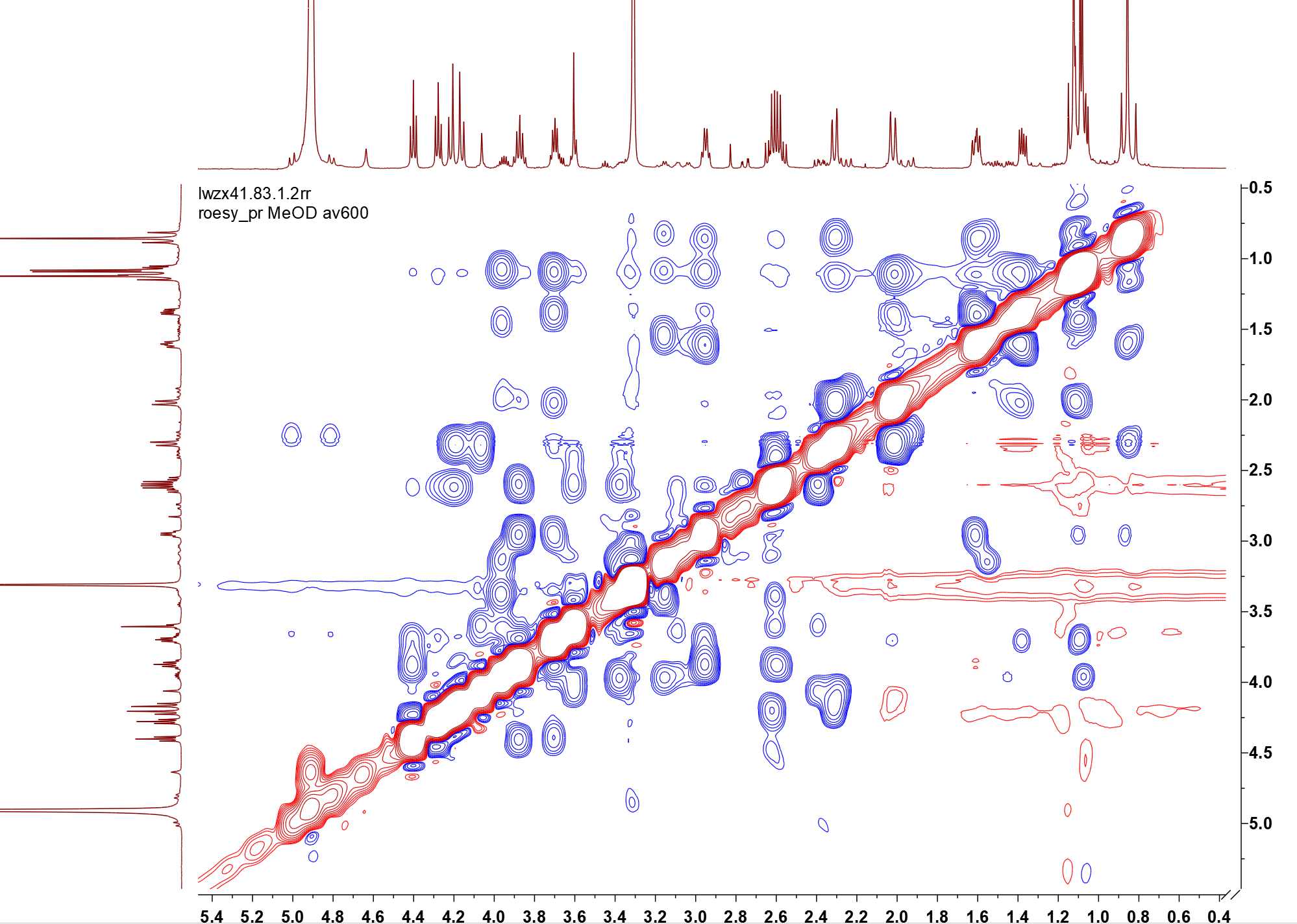


# Figure 23S. (+)-HRESIMS report of **5**.


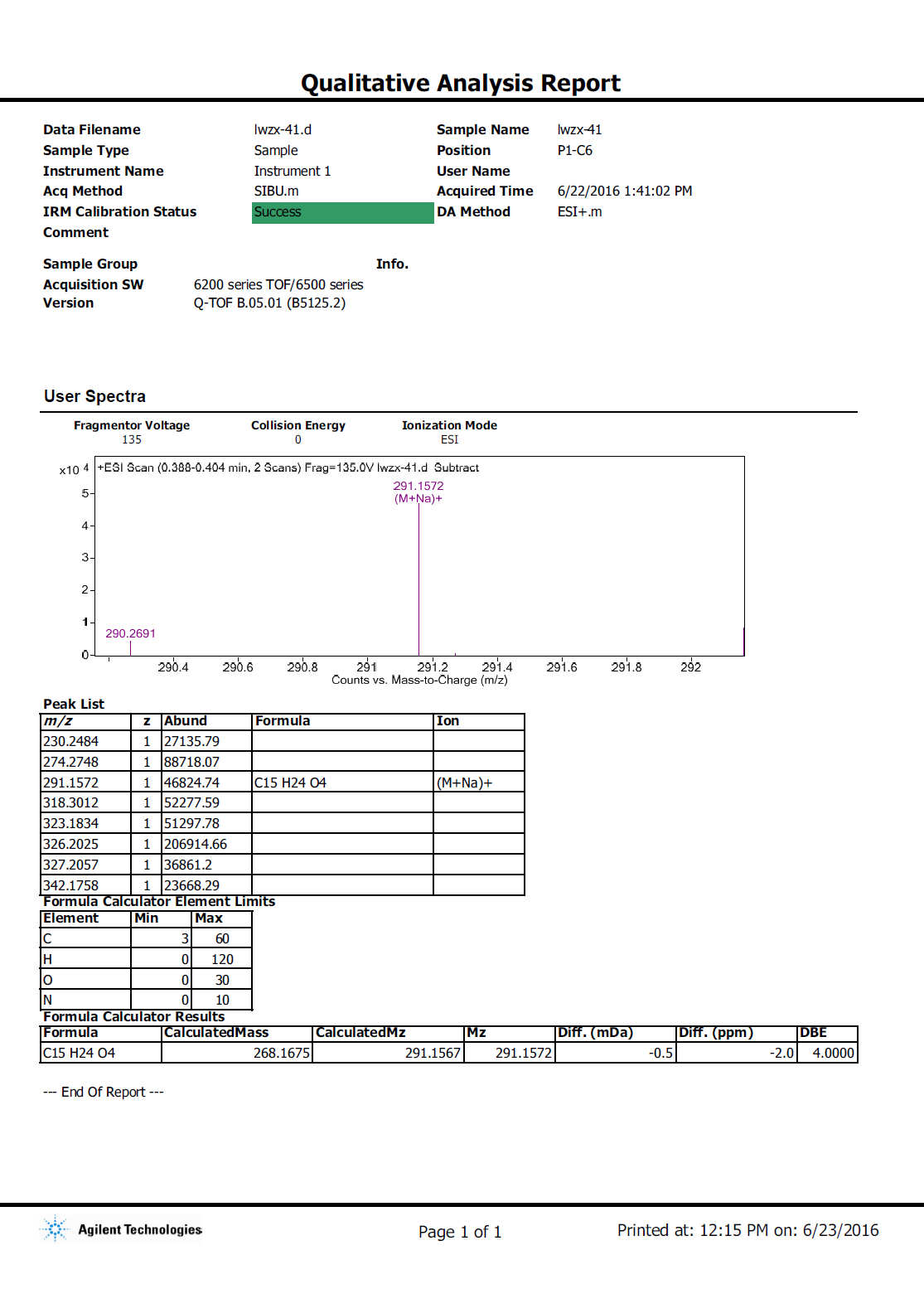


# Figure 24S. ^1^H NMR spectrum of **6** (800 MHz, CDCl_3_).


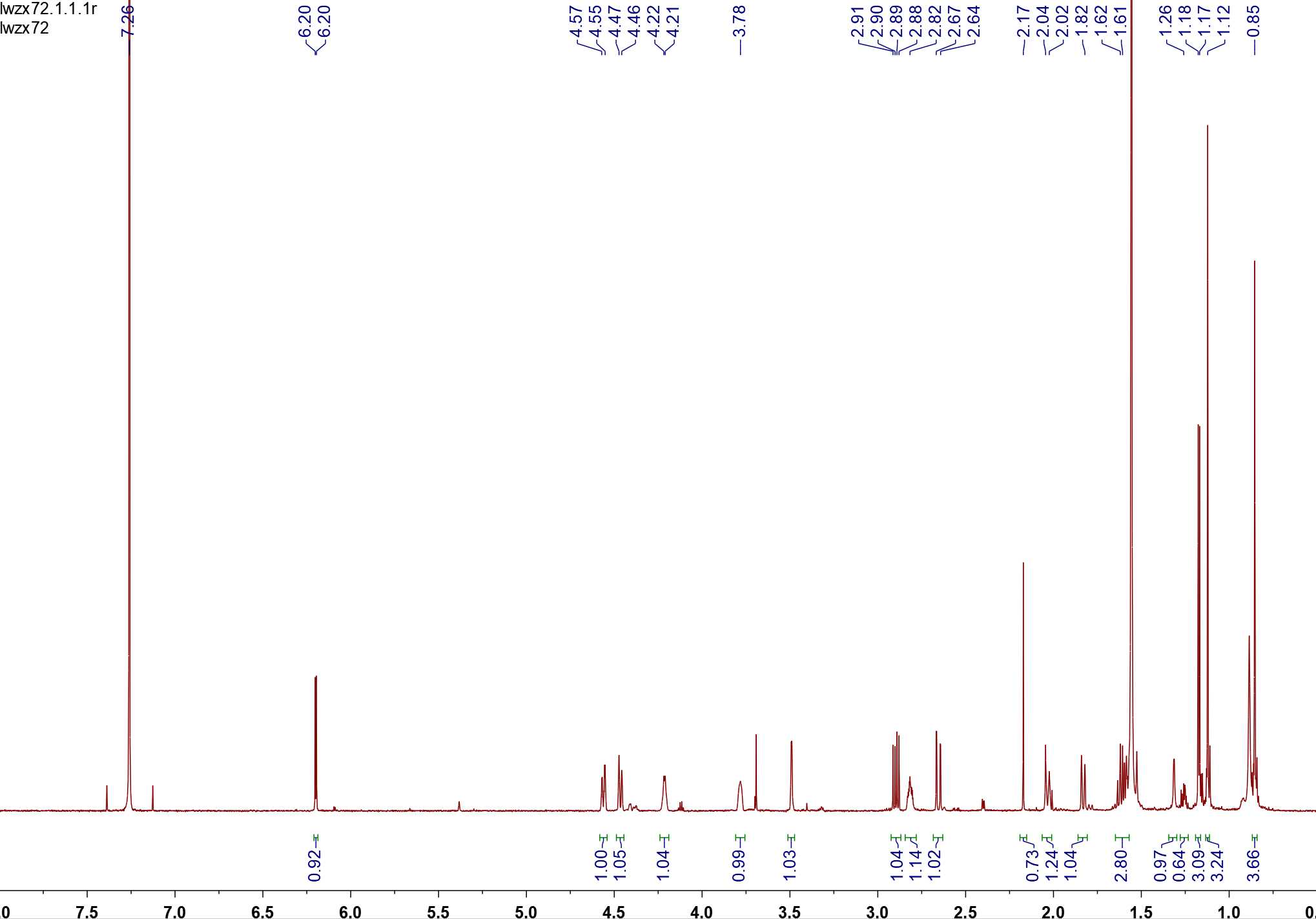


# Figure 25S. ^13^C NMR spectrum of **6** (200 MHz, CDCl_3_).


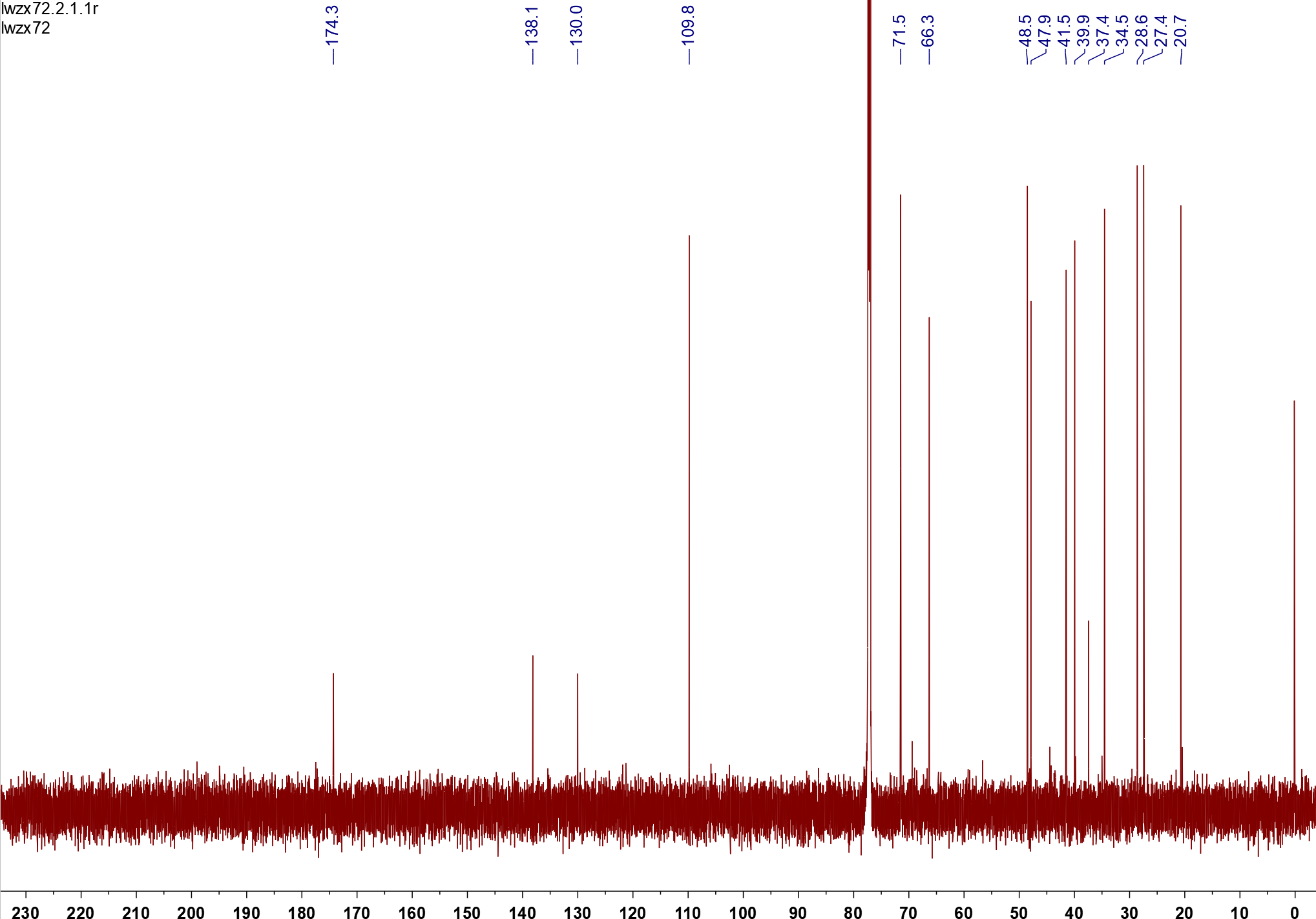


# Figure 26S. HSQC spectrum of **6**.


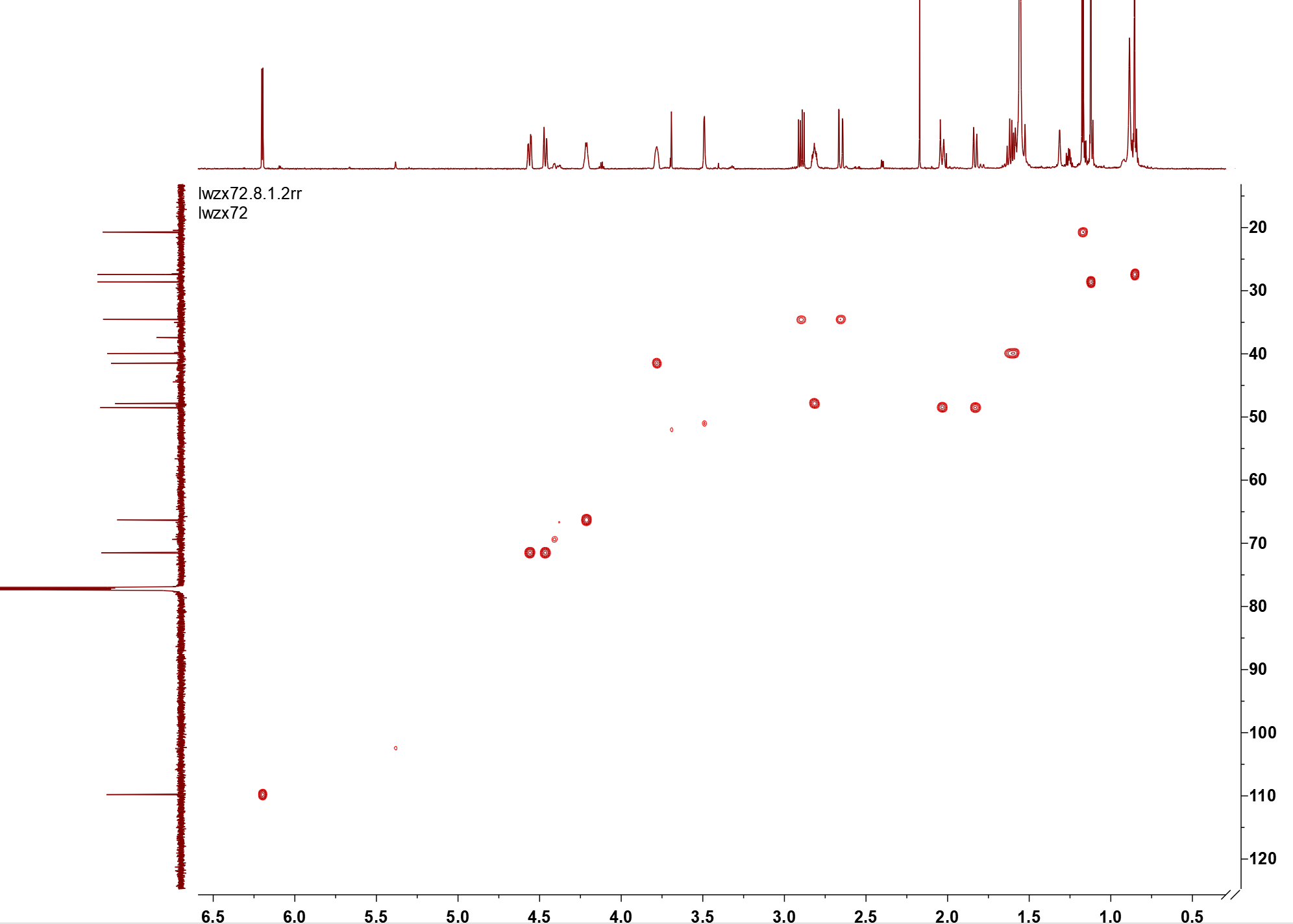


# Figure 27S. ^1^H-^1^H COSY spectrum of **6**.


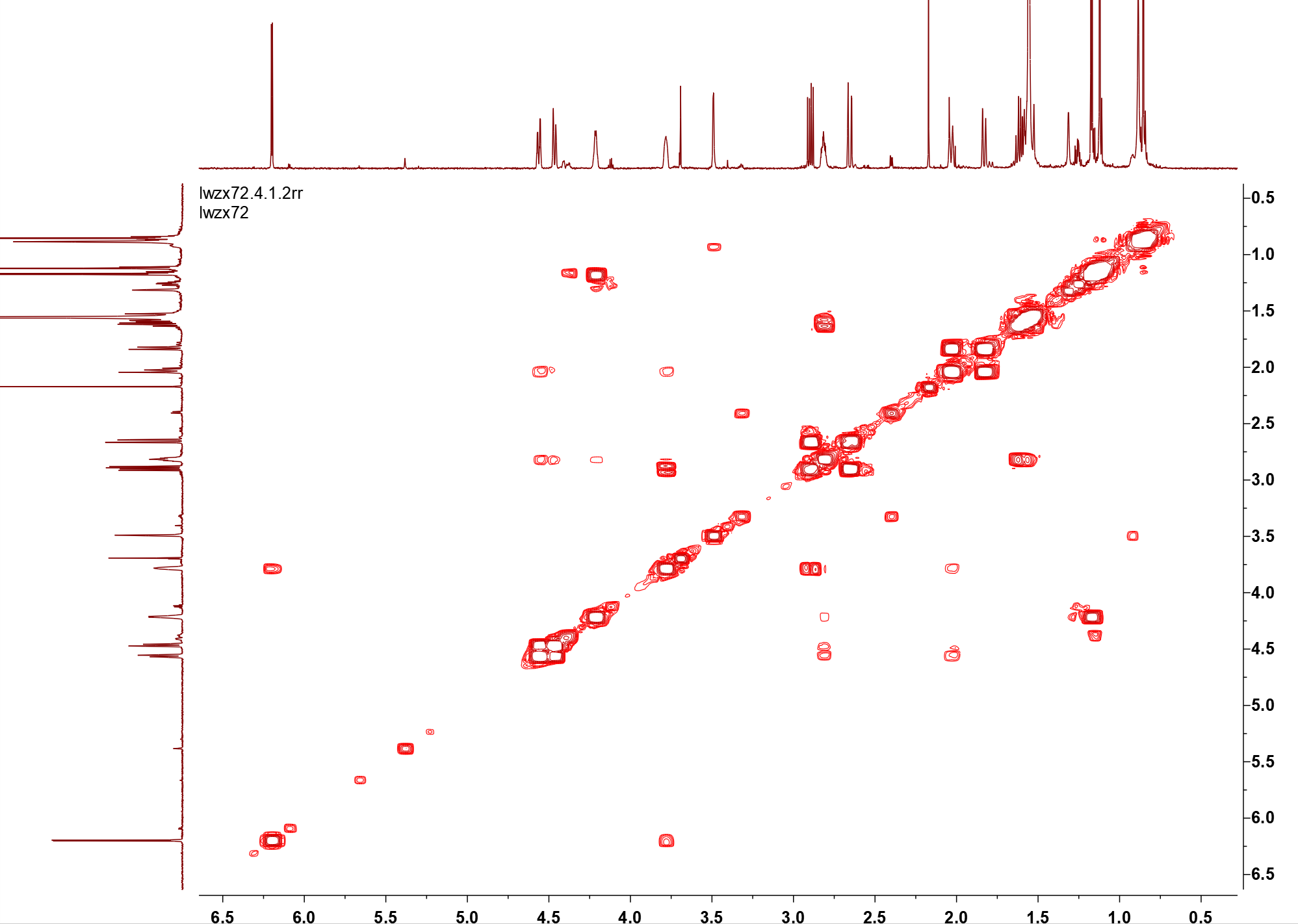


# Figure 28S. HMBC spectrum of **6**.


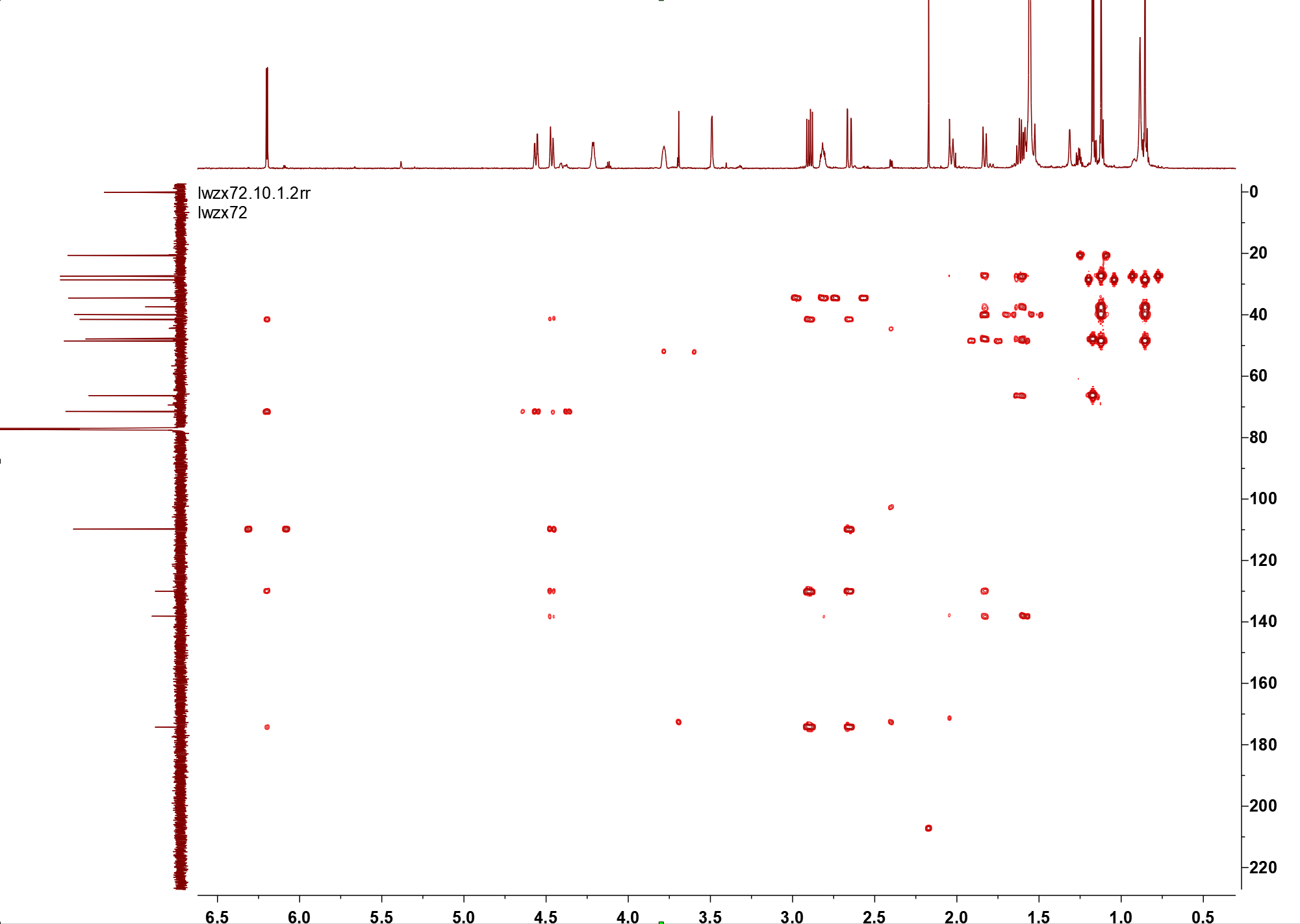


# Figure 29S. ROESY spectrum of **6**.


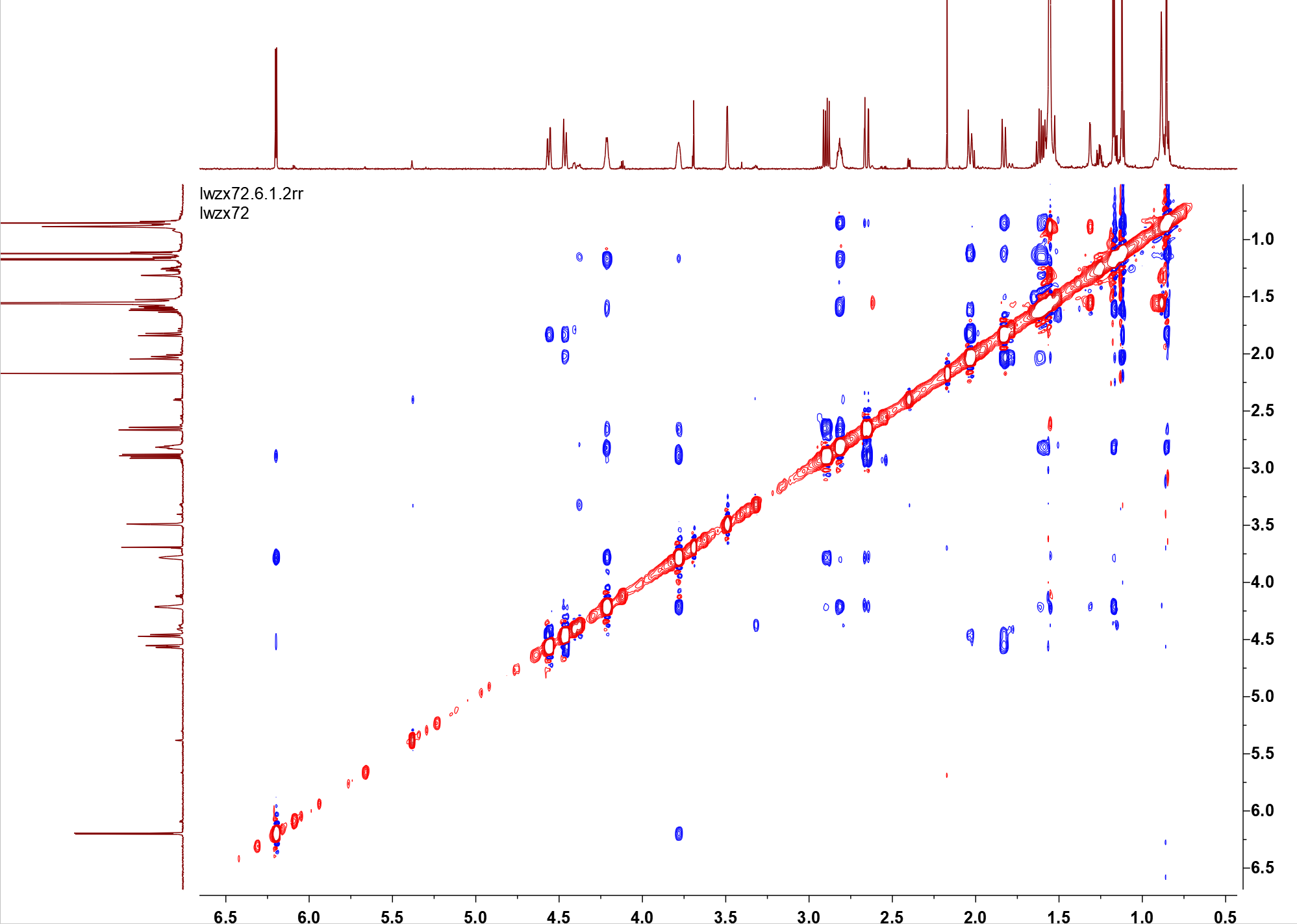


# Figure 30S. (+)-HRESIMS report of **6**.


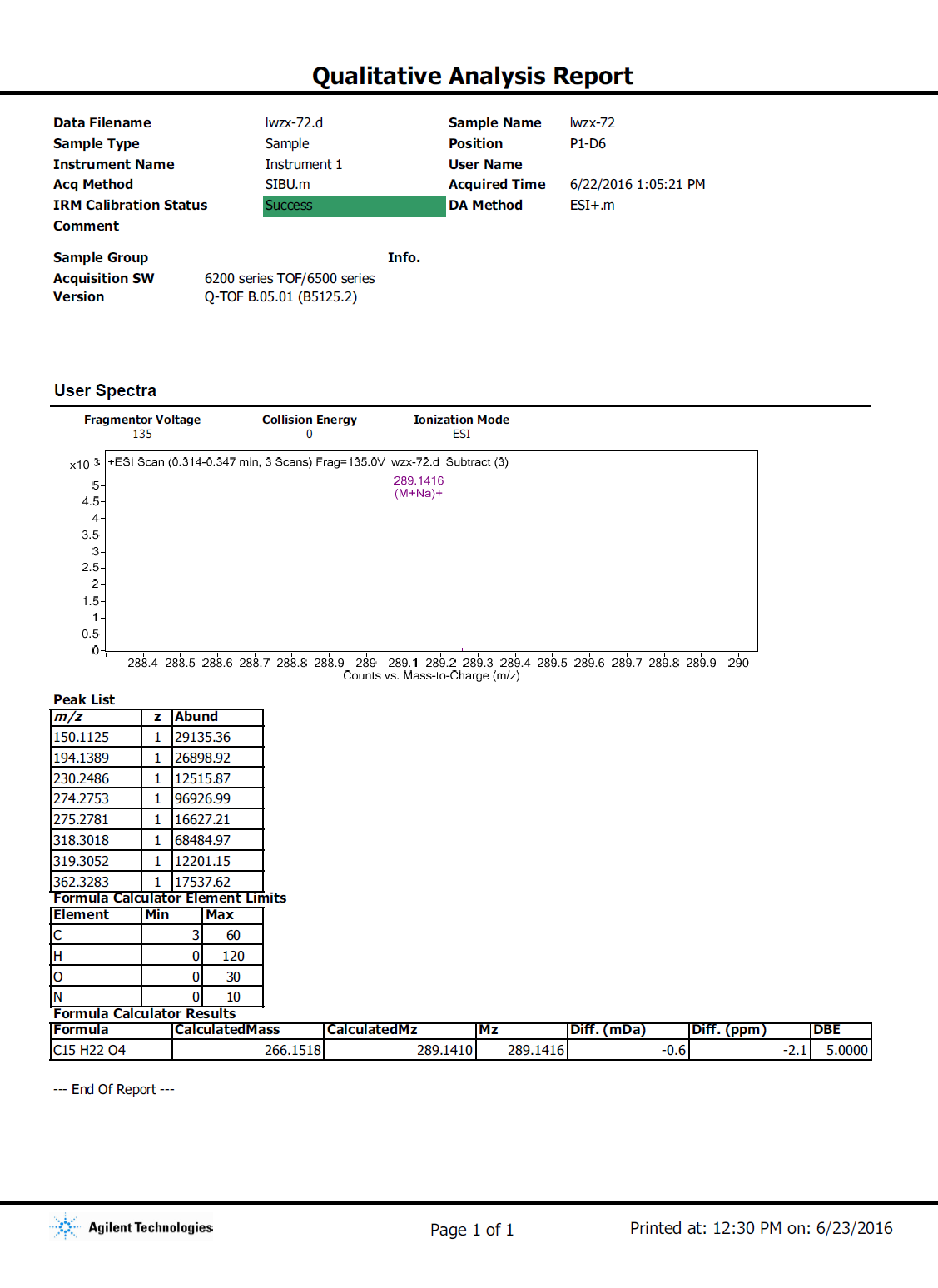

Supplement: Supplementary file 1 — Supplementary material 1 (DOCX 7398 kb) [file 13659_2018_157_MOESM1_ESM.docx]
